# Psoriasis vulgaris leaves a dynamic imprint on circulating and skin γδ TCR repertoires shaped by disease severity, age, and sex

**DOI:** 10.3389/fimmu.2025.1670364

**Published:** 2025-10-17

**Authors:** Maja Jirouš Drulak, Martina Mihalj, Mario Štefanić, Vera Plužarić, Marija Šola, Maja Tolušić-Levak, Marina Marković, Peter Balogh, Stana Tokić

**Affiliations:** ^1^ Department of Medical Chemistry, Biochemistry and Clinical Chemistry, Faculty of Medicine, Josip Juraj Strossmayer University of Osijek, Osijek, Croatia; ^2^ Department of Physiology and Immunology, Faculty of Medicine, Josip Juraj Strossmayer University of Osijek, Osijek, Croatia; ^3^ Department of Dermatology and Venerology, University Hospital Osijek, Osijek, Croatia; ^4^ Department of Nuclear Medicine and Oncology, Faculty of Medicine, Josip Juraj Strossmayer University of Osijek, Osijek, Croatia; ^5^ Department of Laboratory Medicine and Pharmacy, Faculty of Medicine, Josip Juraj Strossmayer University of Osijek, Osijek, Croatia; ^6^ Department of Histology and Embryology, Faculty of Medicine, Josip Juraj Strossmayer University of Osijek, Osijek, Croatia; ^7^ Department of Immunology and Biotechnology, Faculty of Medicine, University of Pecs, Pecs, Hungary

**Keywords:** Psoriasis vulgaris, γδ T cells, TCR repertoire, transcriptome, RNA-Seq, skinimmunity

## Abstract

Psoriasis vulgaris (PV) is a common, T cell mediated dermatosis with substantial systemic footprint. While αβ T cells are well established drivers of PV, the role of γδ T cells, including their abundance, clonal architecture and transcriptional programs in PV remain incompletely understood. To address this, we performed an integrated analysis of circulating and cutaneous γδ cells from 65 patients with PV and 35 healthy controls using TCR repertoire sequencing, bulk transcriptomics, and flow cytometry. In PV, disease severity and age drove contraction of peripheral γδ T cell repertoires, marked by loss of rare clonotypes and hyperexpansion patterns. Subset composition, segment usage, and CDR3 length of both skin and blood clonotypes were further modulated by age, disease severity, and sex, highlighting nuanced repertoire remodeling. TCRγ clonotypes showed partial overlap between blood and skin, whereas TCRδ clonotypes remained private and tissue-specific, with no PV-specific clonotypes identified. Transcriptomic profiling indicated that circulating γδ T cells adopt an activated, cytotoxic, tissue-homing phenotype, consistent with enhanced potential to migrate into and act within lesional skin, especially in a subset of patients. Collectively, these findings demonstrate that PV drives dynamic, clinically modulated remodeling of γδ T cells across compartments, positioning them as dynamic elements of the psoriatic immune landscape and potential targets for future functional and therapeutic investigation.

## 1 Introduction

Psoriasis vulgaris is a chronic erythematosquamous dermatosis of unknown etiology that usually persists for life. The characteristic features of the disease include skin plaques that are often accompanied by rheumatic, intestinal or cardiovascular manifestations of the disease. Both cutaneous and systemic disease features can be ameliorated by immunosuppressive (cyclosporine A, methotrexate) or cytokine-blocking, biologic therapy (anti-TNFα, anti–IL-23p19, anti-IL23p40, anti–IL-17A, and anti-IL-17) (1), alleviating symptoms in most but not all treated patients. Persistent PV has mostly been attributed to IFN-γ and IL-17A-producing αβT cell infiltrate (2–5), but the contributing role of skin-homing γδ T cell subset has been demonstrated as well (6–10). In mouse models of skin inflammation and PV, peripheral γδ T cells colonize challenged dermis in increased numbers (6, 11), mediating enhanced effector functions (6, 7, 12–16) and an exacerbated inflammatory response with systemic effects (12, 17, 18). In humans, γδ T cells are rapidly recruited into physically perturbed skin (8), and their numbers are significantly altered in both the skin (6) and blood of PV patients (9, 10). However, the intrinsic changes that underlie shifts in their phenotypic and functional features or their T-cell receptor (TCR) repertoire in PV are still poorly understood and seldom addressed (10, 19, 20).

In healthy adults, the circulating γδ T cell pool is predominantly populated by Vδ2 expressing cells with conserved Vγ9 chain pairing; in contrast, Vδ2^–^ cells, especially the Vδ1^+^ subset, are enriched across tissues (21). Rare populations expressing Vδ3–8 further complement the diversity of the human Vδ2^-^ T cell compartment, particularly in the liver, jejunum, spleen and lung (21, 22). Vγ9Vδ2 cells exhibit a predominantly innate-like biology with constrained length and limited diversity of the TCR complementarity determining region 3 (CDR3) (23), allowing a low degree of clonotypic plasticity against butyrophilin 3A1 (BTN3A1)-restricted microbial or self-derived phosphoantigens (pAg) (24, 25). On the other hand, Vδ1^+^ lymphocytes harbor individual, mostly private repertoires with greater diversity in CDR3 length and Vγ-chain pairing (26), that typically expand and become more focused in response to acute viral infections (26, 27), reflecting the adaptive nature of their immunobiology. The numerical and clonotypic expansion of the Vδ1^+^ compartment is accompanied by a transition from the naïve (CD27^hi^CD45RA^+^) to the effector memory phenotype (CD27^lo/neg^CD45RA^+^), characterized by increased proliferative capacity and TCR reactivity, altered homing ability, and acquisition of cytotoxic granzymes. These adaptive-like features of the peripheral Vδ1^+^ T-cell compartment develop upon transcriptional reprogramming analogous to antigen-driven CD8^+^ αβT cell differentiation (28), with significant enrichment in genes linked to cytotoxicity, antigen processing and presentation, TCR/co-stimulatory receptors and IFN-γ response. Unlike Vδ1^+^ T cells, the adult Vγ9^+^Vδ2^+^ T lymphocytes acquire the innate-effector programming from early life onward (28), featuring central memory phenotype (CD27^+^CD45RA^-^), and semi-invariant TCR repertoire with largely public CDR3γ9 clonotypes (23, 28, 29).

Changes in the peripheral γδ T cell composition, differentiation and TCR repertoire are also driven by sex (30, 31), age (32, 33) and latent CMV infection (34). In line, significantly lower percentages of γδ T cell have been found in older, healthy male versus healthy female comparisons (31). Healthy aging alone induces a significant shift from naïve to late-stage effector phenotype in CMV^-^, and even more so in CMV^+^ individuals (34), driving numeric (35) and clonal expansion of Vδ1^+^ (27) and less abundant, adaptive-like Vγ9^-^Vδ2^+^ pool (23). The predominant Vγ9/Vδ2 usage in young individuals is, moreover, significantly skewed toward Vγ2/Vδ1 specificity and increased Vγ2–8 gene usage in memory γδ T cell compartment of healthy elderly, underlying significant functional and clonotypic alterations of γδ T cell subsets with age. Similar studies in PV settings are, however, completely missing. To date, reduced numbers of circulating Vγ9Vδ2 T cells expressing skin-homing cutaneous lymphocyte-associated antigen (CLA) (8), and genes controlling IFN-γ/TNF signaling, T cell activation, and proliferation have been found in PV patients (19), and their transition from naïve to terminally differentiated phenotype has been linked to disease severity (19). Previously we reported that the proportions of other, sparsely represented Vδ2^+^ γδTCR^high^ and Vδ1^-^δ2^-^ γδTCR^int^ cells were, in a converse fashion, increased selectively in male PV patients (9), and their peripheral perturbations were linked to altered mRNA/miRNA transcriptional circuits (10). The γTCR repertoire in psoriatic skin lesions has been described as highly polyclonal, private and largely devoid of dominant clonotypes (36), suggesting infiltration of diverse, unexpanded γδ T cell populations. However, the modulating effects of sex, age and CMV serostatus on the abundance, phenotype, or δTCR repertoire of γδ T cells in blood and skin of PV patients, have been largely overlooked.

To address this gap, we performed a bulk-TCRSeq and RNASeq analysis of the peripheral γδ T cell repertoire and immunotranscriptome. Our data reveal that psoriasis drives profound, multifactorial changes in γδ T cell repertoire and transcriptome. These alterations reflect the combined effects of chronic inflammation, aging, disease severity, and sex, contributing to extensive γδTCR repertoire attrition and skewing of the immune landscape toward oligoclonality, potentially impairing immunological responsiveness in psoriatic patients. The discovery of a functionally distinct, pro-inflammatory γδ T cell subset and the identification of public but non-exclusive clonotypes provide a foundation for future research into their roles as biomarkers or therapeutic targets in psoriasis.

## 2 Materials and methods

### 2.1 Subjects

A total of 65 patients with clinically and histopathologically confirmed psoriasis vulgaris (PV) and 35 age- and sex-matched healthy controls were enrolled at the Department of Dermatology and Venereology, University Hospital Osijek, during routine diagnostic procedures. Disease severity and its impact on quality of life were evaluated using the Psoriasis Area Severity Index (PASI) and the Dermatology Life Quality Index (DLQI). The control group comprised unrelated adults with benign, non-infectious, and non-allergic skin conditions. Exclusion criteria included infectious, autoimmune, or malignant diseases, recent allergic reactions (within six weeks before diagnostic procedures), and current systemic or phototherapy treatments. All individuals provided written informed consent.

Peripheral blood and skin biopsy samples were collected along with demographic, anthropometric, and detailed medical history data. Laboratory assessments included complete blood count, inflammatory markers (hsCRP, ESR), lipid profile, and serological markers for *Mycobacterium tuberculosis* (QuantiFERON-TB Gold test), hepatitis B (anti-HBs, HBsAg, anti-HBc IgG/IgM, HBeAg, anti-HBe), hepatitis C (anti-HCV), and cytomegalovirus (anti-CMV IgG/IgM). The study was approved by the Ethics Committee of the Faculty of Medicine in Osijek (Certificate No. 2158-61-07-19-126, October 11, 2019) and the Ethics Committee of the Clinical Hospital Centre Osijek (Certificate No. R2-12487/2019, September 12, 2019).

### 2.2 Peripheral blood mononuclear cell isolation, cryopreservation, and thawing

Peripheral blood mononuclear cells (PBMCs) were isolated from 20 mL of heparinized blood, diluted with 0.9% NaCl (1:1), and layered onto Lymphoprep (Stemcell Technologies, Vancouver, Canada) for density gradient centrifugation (800 x g, 25 min, no brake). The PBMC layer was harvested, washed with PBS, pelleted (400 x g, 10 min) and counted using the LUNA-II Automated Cell Counter (Logos Biosystems, South Korea) with Trypan blue staining. Cells were pelleted again and resuspended in cold cryopreservation medium [10% DMSO, 90% FBS (Sigma Aldrich, Germany)], at 4–5 × 10^6^ cells per aliquot. Cryovials were stored at -80°C in a controlled-rate freezing container (Mr. Frosty, Nalgene) for 48 hours before transfer to liquid nitrogen. For thawing, PBMCs were rapidly warmed in a 37°C water bath and resuspended (1:4) in pre-warmed (37°C) supplemented RPMI-1640 medium [10% FBS, 1% sodium pyruvate (Capricorn Scientific, Germany), and 0.01M HEPES (Sigma Aldrich, Germany)]. The cells were then centrifuged (350 × g, 5 min), resuspended in 5 mL of MACS buffer (PBS, 0.075% EDTA, 0.05% BSA), and analyzed for count and viability using the LUNA-II Automated Cell Counter.

### 2.3 Isolation of total leukocytes from skin

Total leukocytes were extracted from skin tissue using the Whole Skin Dissociation Kit (Miltenyi Biotec, Germany), which combines enzymatic digestion with mechanical dissociation. Skin biopsies were minced and initially passed through a 70 µm strainer to collect free cells. The remaining tissue fragments were then incubated with buffer L and enzymes D and A at 37°C for 3 hours, and afterwards homogenized using the MACS Dissociator (program h_skin_01). The homogenate was filtered through a 70 µm strainer and pooled with the previously collected cells. To maximize cell yield, the original MACS tube was rinsed with cold medium, and the rinse was also filtered into the same collection tube. The pooled suspension was then centrifuged (300 x g, 10 minutes, 4°C), resuspended in 3 mL of medium and counted using the LUNA-II Automated Cell Counter with Trypan blue exclusion. Finally, cells were pelleted again (350 x g, 5 minutes) and cryopreserved at 1 x 10^6^ cells/mL, as described in section 2.2.

### 2.4 γδ T cell immunophenotyping and sorting

Cryopreserved PBMCs were used for γδ T cell immunophenotyping and sorting. γδ T cells were identified using CD3 and pan-γδTCR antibodies, while Vδ2^+^, Vδ1^+^, and Vδ1^-^Vδ2^-^ subpopulations were distinguished using TCRVδ2 and TCRVδ1 antibodies (**Supplementary Table 1**). For immunophenotyping, 0.5 × 10^6^ cells were stained with 0.25 µL of Live/Dead Fixable Near-IR Dead fluorescent dye (ThermoFisher Scientific, USA) for 15 min at 4°C in the dark, washed with PBS, pelleted (350 × g, 5 min), and incubated with 2.5 µL of TruStain FcX (BioLegend, USA) for 10 minutes at room temperature. Antibodies against CD3, γδ TCR TCRVδ1, and TCRVδ2 were then added for 30 minute γδ T cell staining (**Supplementary Figure 1**), followed by two PBS washes (350 × g, 5 min). Samples were acquired on a BD FACS Canto II cytometer (Becton Dickinson, San Jose, CA, USA), with gating optimized using fluorescence-minus-one (FMO) controls, and compensation set with single-stained controls. Data were analyzed using the OMIQ Platform (Dotmatics, USA), and representative gating strategies are shown in **Supplementary Figures 1A, B**. Only samples with > 90% PBMC viability and > 50% skin lymphocyte viability were included (median viability was 98.7% (IQR: 97.28–99.5%) for PBMCs and 69.7 (58.8 – 84.6%) for skin-derived lymphocytes).

For γδ T cell purification, two-way fluorescence-activated cell sorting (FACS) was performed using a Bio-Rad S3e cell sorter (Bio-Rad Laboratories, USA). On average, 13.6 × 10^6^ thawed PBMCs (range: 5.65 × 10^6^ – 24.4 × 10^6^) were processed per sample, yielding a median of 142,591 purified γδ T cells (IQR: 71,655 – 249,979). The staining protocol mirrored that of flow cytometry, except for the viability staining, which was validated using same samples processed in parallel for both methods. Sorted γδ T lymphocytes were collected directly into 500 µL of TRI reagent (Sigma Aldrich, Germany) for immediate RNA extraction with Direct-zol RNA MicroPrep Kit (Zymo Research, USA). RNA quantity was assessed using the DeNovix QFX Fluorometer (DeNovix Inc., USA) and the Qubit RNA High Sensitivity Assay kit. Sorting accuracy and purity were assessed in PBMC samples of five randomly selected blood donors by flow cytometry (DxFLEX, Beckman Coulter, USA), with median purity of the sorted γδ T cell populations exceeding 95% (95.62% (IQR: 89.33% – 96.95%)).

### 2.5 Bulk-RNAseq analysis of peripheral γδ T cell immunotranscriptome

A total of 24 γδ T cell libraries (12 control, 12 patient) were prepared using the AmpliSeq for Illumina Immune Response Panel, following the manufacturer’s protocol (Reference Guide v06, February 2019). In brief, libraries were generated through reverse transcription (20 ng RNA), multiplex PCR amplification of 395 gene targets (19 cycles), FuPa digestion, adapter ligation with AmpliSeq™ UD Indexes, and magnetic bead–based purification (MagSi-NGSPREP Plus, MagnaMedics Diagnostics B.V., Netherlands). Refined libraries were amplified in the second amplification step (7 cycles), followed by two-rounds of library cleanup. Libraries were quantified using KAPA Library Quantification Kit for Illumina platforms (Kapa Biosystems, USA) on a QuantStudio 5 system (ThermoFisher Scientific, USA) and assessed for fragment size (~300 bp) via agarose gel electrophoresis (1.5% gel, 150 V, 25 min). Libraries were diluted to 2 nM, pooled, denatured with 0.2 M NaOH, and sequenced on the Illumina MiniSeq platform (paired-end 2 × 150 bp) with 1% PhiX spike-in. Sequencing quality was assessed using Sequencing Analysis Viewer (SAV) v2.1 (Illumina, USA).

FASTQ reads were imported into the Galaxy platform, where FastQC was used for quality control. Reads shorter than 20 bp or with a quality score below Q20 were removed using Cutadapt. Remaining reads were aligned to the GRCh38 reference genome (UCSC Genome Browser), using the STAR aligner. Gene counts were obtained using FeatureCount, and differential gene expression (DEG) analysis was performed with the *DESeq2* R package. Low-abundance transcripts with fewer than 10 reads in at least five samples per group (PV or HC) were excluded. Gene ontology and gene set enrichment analyses were performed with the clusterProfiler, AnnotationDbi, and msigdbr packages, while results were visualized with ComplexHeatmap, enrichplot, pathview, and ggplot functions.

### 2.6 Bulk-TCRSeq of peripheral blood γδ T cells and skin-derived leukocytes

Libraries targeting CDR3 regions of γ- and δ-TCR chains were prepared using the Archer^®^ Immunoverse™-HS TCR kit (Archer^®^Dx, USA), following the manufacturer’s protocol. A total of 72 RNA samples were used, including 40 from sorted peripheral blood γδ T cells and 32 from skin-derived leukocytes, of which 10 were paired. Library preparation began with TCR-specific RT priming of 50 ng total RNA, followed by two-step cDNA synthesis. Libraries underwent end repair, AMPure bead-based purification, and two-step ligation with MBC adapters containing a P5 index, with unligated adapters removed via dual bead-based purification. Library enrichment included two PCR rounds: the first amplified TCR sequences with gene-specific primers, and the second added P7 indexes, with bead-based (MagSi-NGSPREP Plus) purification between rounds. The final libraries (~300 bp) were validated via electrophoresis, quantified by qPCR (KAPA Library Quantification Kit), normalized to 2 nM and sequenced on the Illumina NextSeq platform (paired-end 2 x 150 bp) with 20% PhiX spike-in.

### 2.7 TCR sequencing quality control and data pre-processing

TCR sequences were aligned, error-corrected, and CDR3 regions reconstructed using MiXCR (v.4.6.0.) (37). To ensure data reliability, four peripheral blood and twelve skin-derived γδTCR libraries were excluded due to low sequencing depth, limited repertoire size, or disproportionate TRA/TRB vs. TRG/TRD read counts. Specifically, excluded samples included one blood library with extremely low read count (26 reads vs. a median of 1,001,089), four skin samples lacking TRG and/or TRD sequences, one blood (7 clonotypes vs. a median of 1,548) and eight skin samples (≤ 10 clonotypes vs. a median of 108.5) with very low TRG/TRD clonotypes, and two blood libraries with disproportionately high TRA/TRB transcript proportions (91.1% and 66.7% vs. a median of 12.5%). Sequencing depth generally did not correlate with clonotype counts, except for a significant correlation between skin TRD read counts and clonotype numbers (ρ = 0.621, P = 0.003), suggesting partial under-sampling of skin TRD repertoires. Detailed sequencing metrics of blood and skin TRG and TRD repertoire are provided in **Supplementary Table 2** -**Supplementary Table 5**.

MiXCR clonotype tables were next converted to VDJTools format, filtered to remove erroneous and non-functional clonotypes, and corrected by merging low-abundance clonotypes with similar, high-abundance ones, reducing sequencing and PCR errors. Pseudogenes (e.g., TRGV10), and TRAV-incorporating variants were also excluded, retaining only functional TRG (TRGV2, TRGV3, TRGV4, TRGV5, TRGV8, TRGV9) and TRD (TRDV1-TRDV8) segments. This filtering preserved > 95% of sequencing reads (blood: 99.1 ± 1.9% of TRD and 97.5 ± 4.1% of TRG; skin: 95.17 ± 6.96% of TRD and 99.7 ± 0.77% of TRG). Final datasets included 1.73 × 10^7^ TRG and 8.85 x 10^6^ TRD blood-derived sequences, and 6.01 x 10^5^ TCRγ and 1.58 x 10^5^ TCRδ cutaneous sequences. The average reads per sample were 4.95 x 10^5^ TRG and 2.53 x 10^5^ TRD in blood, and 2.4 x 10^4^ TRG and 7.89 x 10^3^ in skin (**Supplementary Table 4** and **Supplementary Table 5**). The average number of unique clonotypes in blood samples was 928 (min-max:122–2292) for TRG and 933 (min-max: 76–2176) for TRD, while in skin samples the averages were 73 (19–182) for TRG and 19 (6–56) for TRD (**Supplementary Table 4** and **Supplementary Table 5**).

### 2.8 TCRγ and TCRδ repertoire analysis

TRG and TRD repertoires were subsequently analyzed using VDJTools (38) and the Immunarch R package (39). CDR3γ and CDR3δ clonotypes were classified by TRGV and TRDV variants, focusing on the most prevalent TRDV (TRDV1, TRDV2, TRDV3) and coding TRGV segments (TRGV2, TRGV3, TRGV4, TRGV5, TRGV8, TRGV9). Basic repertoire metrics (read counts, clonotype numbers, CDR3 length), were obtained using the *CalcBasicStats* command. Clonotype frequencies were normalized within each TRGV and TRDV category to sum to 1, ensuring diversity assessments were independent of their overall contribution to the total repertoire. Gene segment usage and CDR3 length distributions were assessed with *CalcSegmentUsage* and *CalcSpectratype*, respectively. V-J pairing frequencies were visualized with *PlotFancyVJUsage*. Diversity indices (D50, Chao1, Efron-Thisted estimate, inverse Simpson and Shannon-Wiener index) were computed using *CalcDiversityStats.* Shared clonotypes were identified using *JoinSamples*, while inter-sample similarity was assessed with Immunarch’s *repOverlap*, calculating the Jaccard index. Additional trends in CDR3 length and gene usage were explored with *repExplore* and *trackClonotypes*, respectively. For age-associated correlation analyses in peripheral repertoires, the four oldest PV patients were excluded to avoid interpretation bias due to the absence of age-matched healthy controls.

## 3 Results

### 3.1 Subject characteristics and γδ T cell immunophenotypic profiles in psoriasis versus healthy controls

The basic characteristics of the study participants are listed in **Table 1**. Psoriasis patients (PV) and healthy controls (HC) were well matched for sex and age, though PV patients had modestly higher BMI. Most PV participants had moderate-to-severe disease with a disease duration of 9 years. In peripheral blood, leukocyte counts were elevated in PV, whereas hsCRP levels did not differ between groups. CMV seroprevalence was high in both groups, consistent with trends in Croatian population (40), though rates were slightly lower in PV. In contrast, anti-HBs seropositivity was markedly reduced in PV compared to HC (20/42 vs. 16/14; *P* = 2.2 × 10^–16^), likely reflecting differences in prior vaccination under Croatia’s mandatory HBV immunization program. Flow cytometry revealed no significant case-control differences in the overall frequency of circulating γδ T cells, or their major subsets (Vδ1^+^, Vδ2^+^, or Vδ1^-^Vδ2^-^). However, sex-stratified analysis revealed a selective reduction in total γδ T cells and the Vδ2^+^ subset in male PV patients compared to healthy male controls (**Supplementary Figure 2**). This finding is consistent with prior reports of diminished peripheral Vγ9Vδ2 T cells in psoriasis (8, 19), but here points to a male-specific effect. However, healthy female controls in our cohort displayed inherently lower proportions of γδ T (median 3.11% [2.48 – 3.91] vs. 5.18% [3.07 – 7.46] in males, P = 0.028) and Vδ2^+^ cells (1.57% [1.37 – 2.42] vs. 3.37 [1.93 – 6.68] in males, P = 0.018) compared to healthy males, potentially masking disease-associated alterations in unstratified comparisons. Frequencies in female PV patients did not differ significantly from healthy females or affected males, suggesting sex-specific γδ T cell dynamics that warrant further study in larger, sex-balanced cohort. In psoriatic lesions, CD3^+^ T cells were significantly enriched compared to healthy skin (**Supplementary Figure 3A**), consistent with infiltration of T cells in inflamed skin. Within the γδ T cell compartment, the Vδ1^–^Vδ2^–^ subset was significantly expanded in PV skin (**Supplementary Figure 3B**). Intriguingly, female patients showed a progressive expansion of Vδ2^+^ cells with increasing PASI scores (ρ = 0.846, P = 9.7 x 10^-4^), while overall γδ T cell frequencies in psoriatic skin declined sharply with age (ρ = -0.722, P = 9.83 x 10^-8^), accompanied by reduced frequencies of the Vδ1 subset (ρ = -0.401, P = 0.009). These associations were absent in healthy participants, suggesting disease-associated remodeling of γδ T cell subsets.

**Table 1:** Baseline subject characteristics and distribution of γδ T cell subsets in blood and skin.

	PV	HC	P
Age	44 (33 – 57)	37 (31 – 47)	0.137*
Sex (M/F)	49/16	25/10	0.812**
BMI	30.12 (25.23 – 32.79)	27.14 (25.82 – 29.33)	0.048*
hsCRP (mg/L)	1.60 (0.68 – 3-91)	1.38 (0.70 – 2.40)	0.603*
L (N x10^9^/L)	7.4 (6.40 – 9.50)	6.4 (5.15 – 7.05)	0.001*
CMV IgG (U/mL)	97.85 (45.45 – 129)	108 (89.95 – 130)	0.214*
CMV IgG (pos/neg)	49/13	29/1	0.031**
PASI	16.1(7.15– 22.25)	–	–
Disease duration (years)	9 (5 – 17)	–	–
% γδTCR^+^ of CD3^+^ in PB	6.85 (3.96 – 9.5)	6.34 (4.75 – 8.23)	0.764*
% Vδ2^+^ of γδ T in PB	68.8 (51.4 – 85.4)	66.4 (48.2 – 81.3)	0.667 *
% Vδ1^+^ of γδ T in PB	21.1 20.4 (10.75– 32.58)	26.2 (14.3 – 41.6)	0.251*
%Vδ1^-^Vδ2^-^ of γδ T in PB	4.39 (2.32 – 12.9)	3.92 (2.18 – 11.4)	0.598*
% γδTCR^+^ of CD3^+^ in skin	1.25 (0.77 – 1.62)	1.76 (1.20 – 2.58)	0.065*
% Vδ2^+^ of γδ T in skin	23.5 (16.3 – 36.9)	22.7 (14.13 – 53.08)	0.669*
% Vδ1^+^ of γδ T in skin	36.6 (29 – 60.5)	46.1 (31.33 – 61.03)	0.461*
%Vδ1^-^Vδ2^-^ of γδ T in skin	23.7 (18.1 – 38.9)	17.7 (8.96 – 27.5)	0.029*

Continuous data are shown as median (interquartile range). BMI, body mass index, hsCRP, high sensitivity C, reactive protein, L, leukocyte count, CMV, cytomegalovirus; PASI, psoriasis area and severity index; PB, peripheral blood. * Mann-Whitney U test, ** Fisher’s Exact Test. Bold values indicate P < 0.05.

### 3.2 Distinct alterations in γδ TCR gene segment usage between blood and skin of psoriatic patients

**Figure 1:**
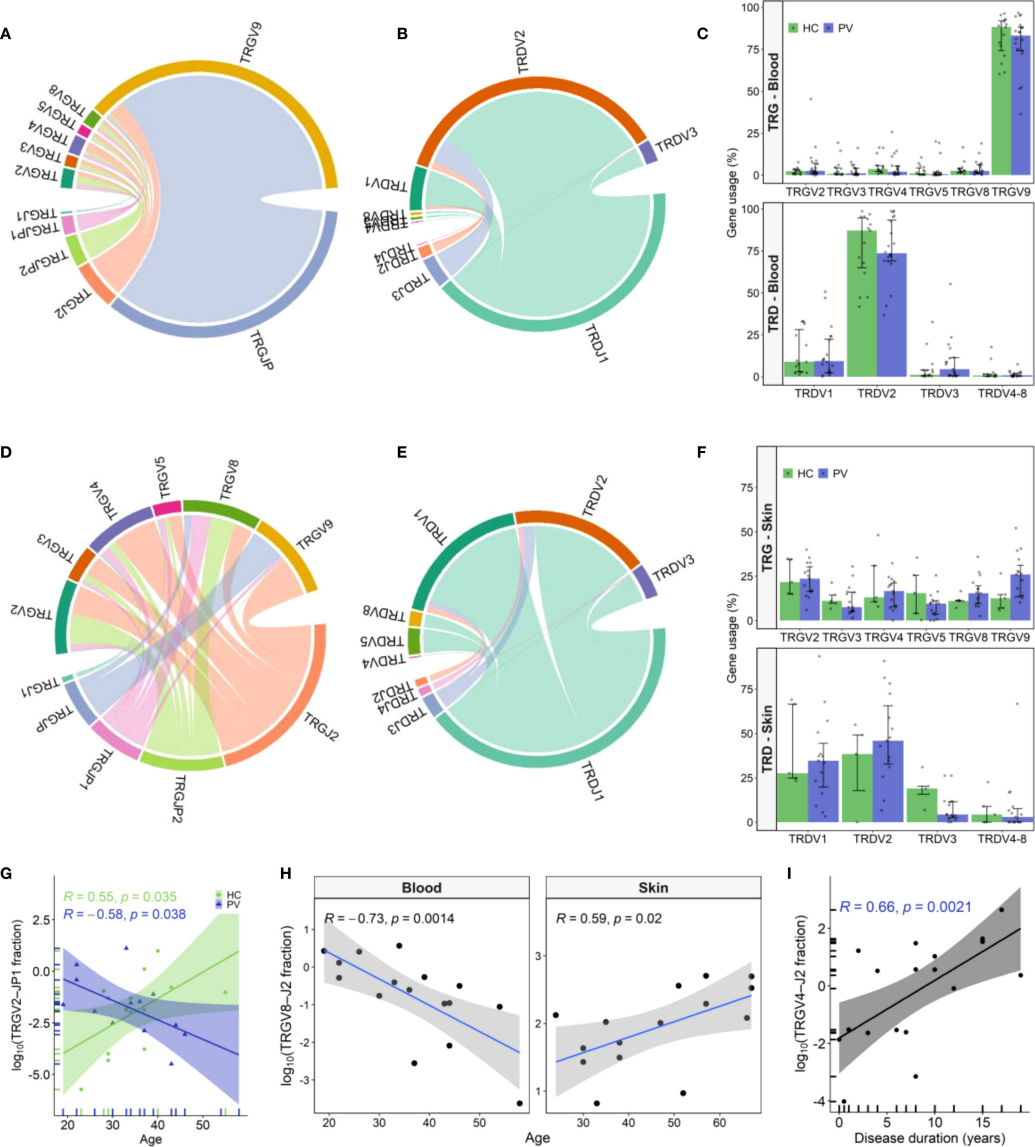
TRG and TRD gene usage patterns in peripheral blood and skin of psoriasis patients and healthy controls. **(A, B)** Chord diagrams from pooled peripheral blood γδ T cell clonotypes from psoriasis vulgaris (PV; N = 20) and healthy controls (HC; N = 15) depict TRGV–TRGJ **(A)** and TRDV-TRDJ **(B)** rearrangements, with TRGV9-TRGJP and TRDV2-TRDJ1 being the most prevalent pairings. **(C)** TRGV and TRDV segment usage in blood did not differ between PV and HC. Bars represent median ± IQR. **(D, E)** Chord diagrams from skin-derived clonotypes (PV: N = 15; HC: N = 5) demonstrate greater TRGV-TRGJ **(D)** and TRDV-TRDJ **(E)** diversity compared to blood, with increased usage of non-TRGV9 segments and a more balanced TRDV1/TRDV2 distribution. **(F)** In skin, TRGV9 usage is elevated and TRDV3 reduced in PV relative to HC, but differences did not remain significant after correction for multiple testing. Bars represent median ± IQR. **(G)** Frequency of circulating TRGV2-TRGJP1 clonotypes declines with age in PV patients (N = 26) but increases with age in HC (N = 15). **(H)** TRGV8-TRGJ2 clonotypes decrease with age in blood of PV patients (N = 26) but increase in lesional skin (N = 15). **(I)** Circulating TRGV4-TRGJ2 clonotype frequency correlates positively with disease duration (N = 30). In all correlation plots, Spearman correlation coefficients (R) and p-values are indicated. In age correlation analyses **(G–H)**, the four oldest PV participants were excluded due to lack of age-matched HC individuals.

The composition of blood and skin γδ TCR repertoires was first assessed by analyzing TRGV/TRGJ and TRDV/TRDJ gene segment usage. As expected, TRGV9 and TRDV2 segments dominated the peripheral blood, accounting for over 80% of clonotypes. The most prevalent variants featured canonical TRGV9-TRGJP and TRDV2-TRDJ1 rearrangements, aligning with prior findings [1–3]. While these clonotypes dominated most blood samples, alternative TRG and TRD rearrangements were also observed across individuals (e.g. TRGV9-TRGJ2, TRGV4-TRGJ2, TRDV1-TRDJ1, TRDV2-TRDJ3), in some cases at frequencies comparable to canonical pairings (**Figures 1A, B**, **Supplementary Figures 4A, B**). However, these variations in the peripheral blood γδTCR repertoire were not associated with disease status, as no significant differences were observed in TRG or TRD gene segment usage between healthy and psoriatic individuals (**Figure 1C**, **Supplementary Table 6** and **Supplementary Table 7**).

The cutaneous TCRγ repertoire displayed a more balanced distribution among TRGV2-TRGV9 variants (**Figure 1D**, **Supplementary Figure 4C**), contrasting the dominance of TRGV9-TRGJP clonotypes in blood. In skin, TRG clonotypes preferentially rearranged with non-TRGJP variants, with TRGJ2 being the most abundant, followed by TRGJP2 and TRGJP1. Similarly, while TRDV2 clonotypes dominated the peripheral blood, the cutaneous repertoire displayed a more equal representation of TRDV1 and TRDV2, with a smaller contribution from TRDV3 clonotypes (**Figure 1E**, **Supplementary Figure 4D**). Notably, psoriatic lesional skin displayed nominal TRGV9 enrichment (**Figure 1F** 25.94% vs. 12.6%, P = 0.034), and TRDV3 depletion (**Figure 1F**; 4.34% vs. 20.28%, P = 0.038) compared to healthy skin; however, these trends did not remain statistically significant following multiple testing correction (Benjamini-Hochberg, **Supplementary Table 8** and **Supplementary Table 9**). Beyond group comparisons, repertoire features were associated with disease severity and age. In lesional skin, TRGV8 clonotype proportions decreased significantly with increasing disease severity (ρ = -0.736, P = 0.003), while TRGV2-TRGJP1 transcripts positively correlated with PASI (ρ = 0.85, P = 0.006). Conversely, in peripheral blood, TRGV2-TRGJP1 clonotypes declined with patient age (**Figure 1G**), paralleling the age-related decline in blood TRGV8-TRGJ2 clonotypes (**Figure 1H**). Notably, this trend was reversed in diseased skin, where TRGV8-TRGJ2 clonotypes increased significantly with age (**Figure 1H**). Additionally, blood TRGV4 clonotypes showed a strong positive correlation with disease duration (ρ = 0.63, P = 0.003), driven primarily by the TRGV4-TRGJ2 rearrangement (**Figure 1I**). These age- and disease-associated shifts in γδTCR repertoire were absent in healthy individuals, who instead exhibited an age-associated increase in TRGV2-TRGJP1 clonotypes (**Figure 1G**).

Collectively, our findings reveal that psoriasis drives distinct alterations in the γδTCR repertoires of peripheral blood and skin, characterized by nominal TRGV9 enrichment and TRDV3 depletion in the skin, along with contrasting disease and age-related patterns in TRGV2-TRGJP1 and TRGV8-TRGJ2 transcript abundance between these compartments compared to healthy controls.

### 3.3 Age, sex, and disease severity influence divergent CDR3 length dynamics in PV

We further evaluated the structural and functional properties of TCRγ (TRG) and TCRδ (TRD) repertoires, including CDR3 length distribution, convergence, and junctional diversity (nucleotide insertions, deletions, and nontemplated additions).

**Figure 2:**
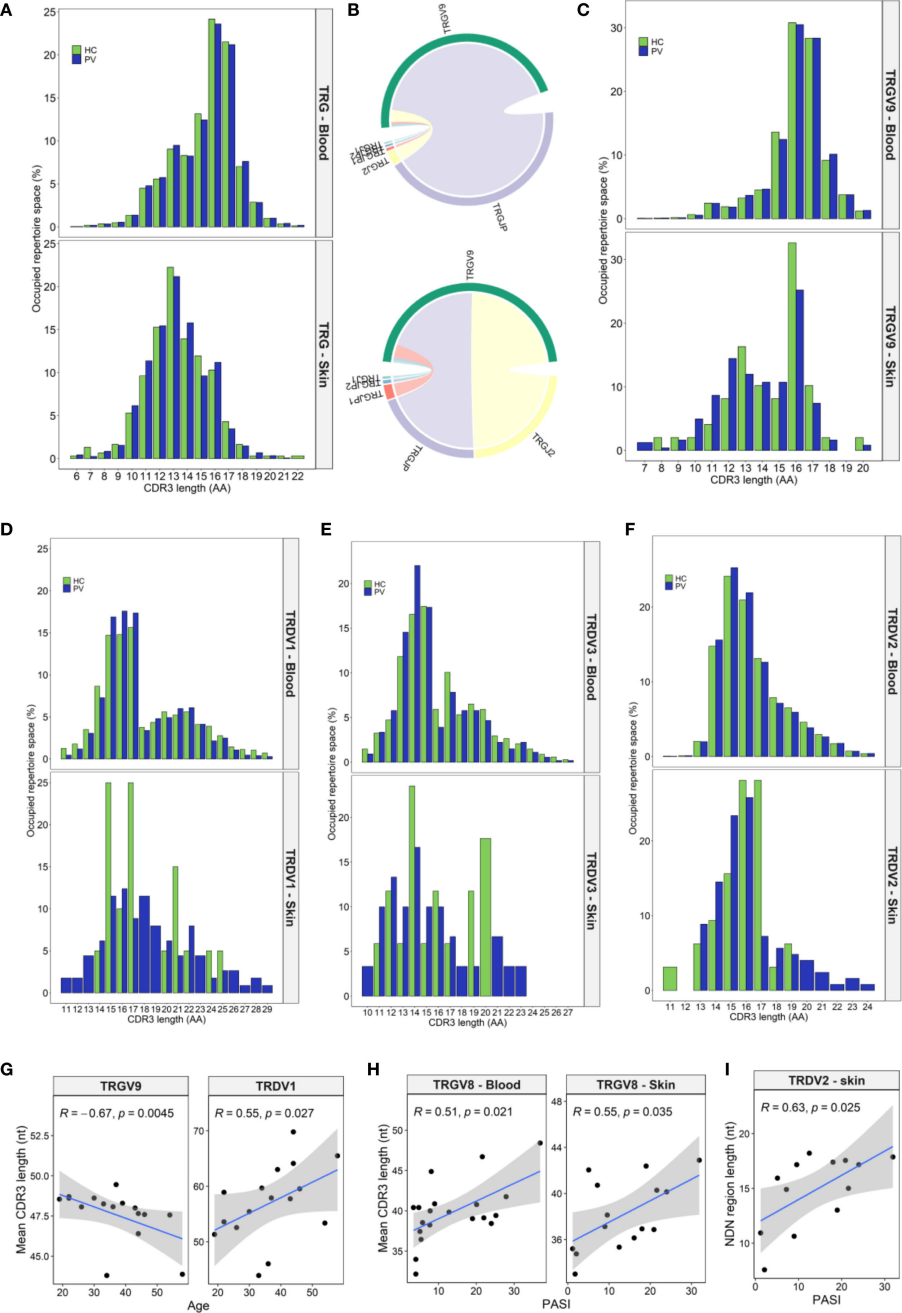
Tissue-specific and psoriasis-associated alterations in CDR3 length distributions of TRG and TRD clonotypes. **(A)** Spectratype analysis of TRG CDR3 length distributions in peripheral blood (PV: N = 20; HC: N = 15) and skin (PV: N = 15; HC: N = 5) reveals that circulating repertoires span a broader range (13–17 amino acids), while skin clonotypes show a narrower and shorter profile (11–16 aa). **(B)** Chord diagrams from pooled blood (N = 35) and skin (N = 15) TRGV9 clonotypes show dominant TRGV9–TRGJP pairings in blood and more balanced TRGV9–TRGJP and TRGV9–TRGJ2 usage in skin. **(C)** This recombinatorial difference underlies the bimodal CDR3 length distribution observed in skin TRGV9 clonotypes. **(D-F)** TRDV1 **(D)** and TRDV3 **(E)** display bimodal length patterns, while TRDV2 **(F)** demonstrates a continuous distribution. Longer CDR3 sequences within the TRDV repertoire are selectively depleted in healthy skin compared to lesional psoriatic skin. **(G)** In PV patients, TRGV9 CDR3 length in blood inversely correlates with age while TRDV1 CDR3 length increases with age. **(H)** TRGV8 CDR3 length positively correlates with Psoriasis Area and Severity Index (PASI) in both blood and skin. **(I)** Increased junctional (NDN) region length in skin TRDV2 clones also associates with higher PASI scores. Data in panels **(A, C)**, and **(D-F)** presents median percentage of repertoire occupancy per CDR3 length bin. Panels **(G–I)** display Spearman correlation coefficients (R) with p-values. In age-related correlation analyses **(G)**, four oldest PV donors were excluded due to lack of matched healthy controls.

TRG clonotypes displayed a wide CDR3 length distribution ranging from 6 to 22 amino acids (**Figure 2A**). Among blood-derived clonotypes, roughly 70% measured between 13 and 17 amino acids (**Figure 2A**), whereas skin-derived clonotypes were significantly shorter, mostly falling within the 11–16 amino acid range (**Figure 2A**), indicative of tissue-specific differences in V–J gene usage. Circulating repertoires were primarily composed of TRGV9-expressing clonotypes (**Figure 1A**), which tend to have longer CDR3 regions (10–20 amino acids, **Figure 2C**), whereas skin repertoires were enriched for TRGV2/3/4/5/8 segments (**Figure 1D**), which typically generate shorter CDR3s (11–15 amino acids, **Supplementary Figure 5**). In addition, TRGV9 clonotypes in the skin primarily featured TRGV9–TRGJ2 rearrangements (**Figure 2B**), in contrast to the TRGV9–TRGJP rearrangements that dominated in the blood (**Figure 2B**), likely contributing to the shorter and bimodal TRGV9 CDR3 length distribution observed in the skin (**Figure 2C**). Conversely, TRGV2/3/4/5/8 lengths remained consistent across both compartments (**Supplementary Figure 5**). Bimodal distributions were also evident in the TRDV1 and TRDV3 (**Figures 2D, E**) repertoires from both blood and skin, whereas TRDV2 clonotypes exhibited a continuous CDR3 length range (**Figure 2F**). Of interest, longer TRDV1/2/3 clonotypes were largely absent in healthy skin compared to lesional skin (**Figures 2D-F**).

Although overall CDR3 length profiles did not differ dramatically between psoriasis patients and healthy controls, subgroup analyses revealed diverse trends in PV that were absent in HC. In patient blood, TRGV9 CDR3 lengths contracted with age, whereas TRDV1 lengths expanded (**Figure 2G**). Female PV patients exhibited longer CDR3δ2 regions than male PV patients (**Supplementary Figures 6A, B**) a sex difference not seen in HC (P = 0.845). In addition, their CDR3δ2 were also longer than those of healthy females (51.04 [50.64–51.24] nt vs. 49.28 [49.21–49.56] nt, P = 0.011), likely reflecting higher frequency of expanded TRDV2–TRDJ3 clonotypes in female patients, which are intrinsically longer than TRDJ1 or TRDJ2 counterparts (**Supplementary Figures 6C, D**). These sex-linked shifts in peripheral CDR3 length profile were not mirrored in skin. Disease severity positively correlated with TRGV8 CDR3 length in blood and skin (**Figure 2H**), and with increased nucleotide insert size in cutaneous TRD clones (ρ = 0.578, P = 0.026), particularly within TRDV2 clonotypes (**Figure 2I**). Finally, TCR convergence, defined as distinct nucleotide sequences encoding identical CDR3 amino acid sequence, declined with age in PV blood for both γ (ρ = −0.55, P = 0.028) and δ chains (ρ = −0.69, P = 0.003), but not in age-matched HC.

Taken together, these results highlight that psoriasis is associated with subtle, yet significant remodeling of γδTCR structural and junctional diversity, modulated by age, disease severity, and sex.

### 3.4 Disease severity drives γδ TCR repertoire contraction in psoriasis

To characterize the γδTCR repertoire diversity, we analyzed clonotype counts, diversity indices, and repertoire space distribution in blood and skin from PV patients and healthy controls. Blood clonotypes were grouped by frequency into ‘hyperexpanded’ (>5%), ‘large’ (0.5–5%), ‘medium’ (0.05–0.5%), and ‘small’ (<0.005%) compartments. In skin, the much lower read depth and clonotype counts (**Supplementary Table 5**) necessitated a simplified scheme: TRG clonotypes were assigned to ‘hyperexpanded’, ‘large’, or ‘medium’ groups, while TRD clonotypes were too sparse for frequency-based partitioning. Repertoire diversity was in addition evaluated using the Efron-Thisted and Chao1 estimators (to capture rare variants), Shannon-Wiener and Inverse Simpson indices (to assess overall richness and evenness), and the D50 index (to quantify clonal expansion).

**Figure 3:**
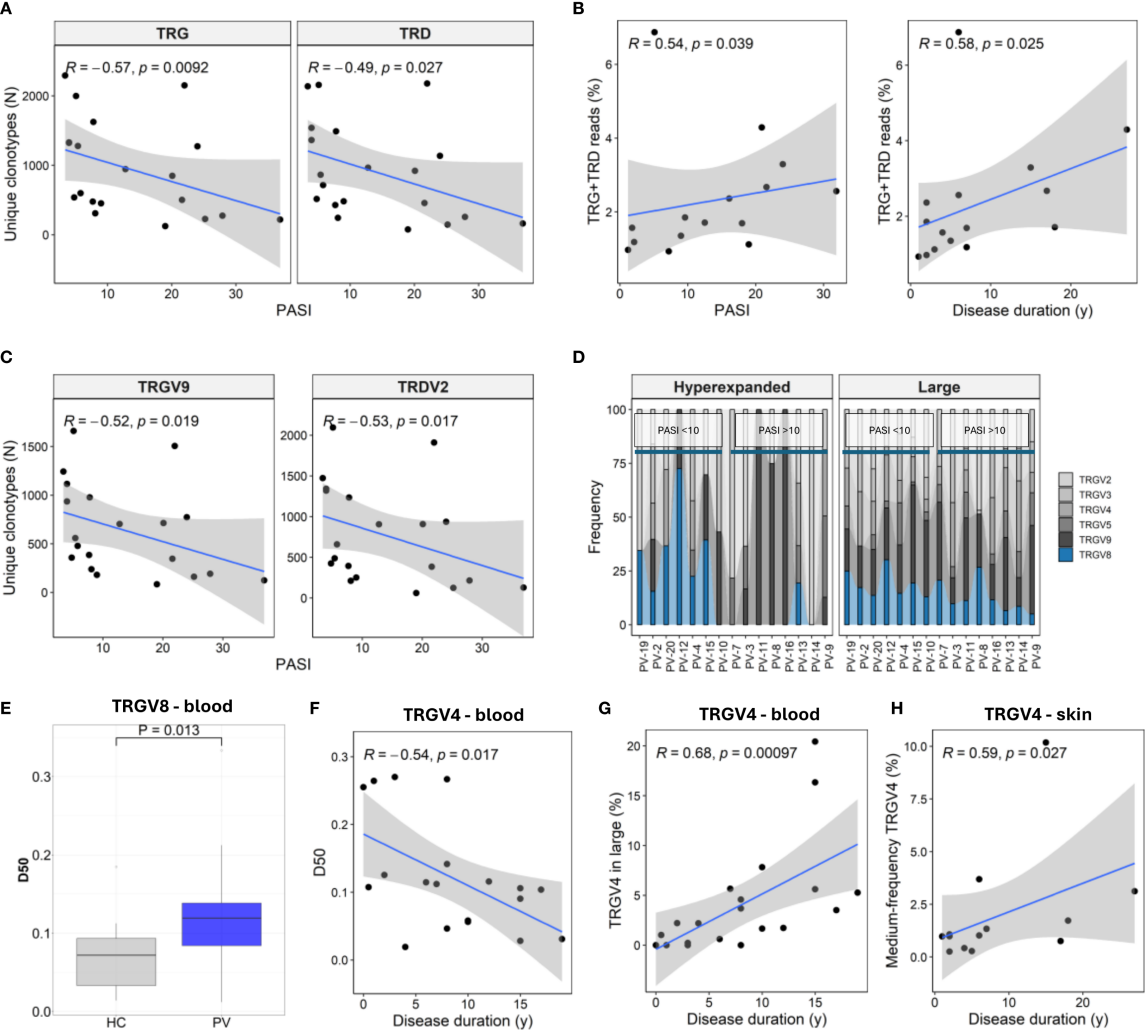
Disease severity and duration shape TRG and TRD repertoires in blood and skin of psoriasis patients. **(A)** Circulating γδ T cell repertoire diversity declines with increasing Psoriasis Area and Severity Index (PASI), as reflected in reduced numbers of unique TRG and TRD clonotypes (N = 30). **(B)** In lesional skin (N = 15), the proportion of TRG and TRD reads increases with both PASI and disease duration. **(C)** TRGV9 and TRDV2 clonotypes in peripheral blood (N = 30) show marked contraction with higher PASI scores. **(D)** TRGV usage within hyperexpanded and large frequency clonotype compartments in lesional skin shows decreased TRGV8 representation in patients with high PASI scores (PASI >10). **(E)** D50 values (fraction of clonotypes required to account for 50% of total reads) of TRGV8 clonotypes are elevated in PV patients. P-value calculated by Mann-Whitney U test. **(F)** TRGV4 repertoire diversity in blood declines with disease duration. **(G)** TRGV4 representation increases within the large frequency clones (0.5 – 5%) in blood. **(H)** The proportion of medium-frequency (0.5 – 5%) TRGV4 clonotypes increases with disease duration in lesional skin. Panels **(A–C, F–H)** show Spearman correlation coefficients (R) with associated p-values.

Overall, total blood TCRγ and TCRδ clonotype counts and diversity indices were similar between PV patients and healthy controls (**Supplementary Table 10**). However, increasing PASI scores were associated with a marked contraction of circulating γ and δ clonotypes (**Figure 3A**), contrasting increasing fraction of cutaneous TRG/TRD reads with both disease duration and severity (**Figure 3B**). The most pronounced PASI-dependent decreases in the blood repertoire were observed for the TRGV9 and TRDV2 subsets (**Figure 3C**). This loss of blood repertoire richness was driven by depletion of rare (‘small’) clonotypes and was mirrored by reduced Chao1 and Efron-Thisted diversity estimates (**Supplementary Table 11**). Concurrently, the ‘hyperexpanded’ TRG compartment increased (**Supplementary Table 11**), indicating a PASI-associated shift toward clonal expansion. Peripheral diversity losses associated with PASI were also observed across TRGV2, TRGV4, TRGV8, and TRDV3 subsets (**Supplementary Table 11**), highlighting that disease severity impacts multiple γδ T cell subsets.

In lesional skin, TRGV8 representation within ‘large’ and ‘hyperexpanded’ fractions declined with PASI (**Figure 3D**), paralleling elevated peripheral TRGV8 D50 values (**Figure 3E**). Disease duration further remodelled the repertoire architecture. In blood, TRGV4 clonotypes showed lower D50 over time (**Figure 3F**), with concomitant enrichment in the ‘large’ frequency compartment (**Figure 3G**), while the medium-frequency TRGV4 clonotypes increased in the skin (**Figure 3H**). Similarly, prolonged disease reshaped the ‘small’ TRD compartment in blood, marked by TRDV2 depletion (ρ = –0.46, P = 0.041) and reciprocal TRDV1 enrichment (ρ = 0.48, P = 0.033).

Collectively, these findings demonstrate that PV severity selectively contracts the peripheral γδ TCR repertoire while reshaping lesional skin subsets, pointing to compartment-specific alterations that may contribute to local inflammation and systemic immune modulation.

### 3.5 Age-driven peripheral γδTCR contraction outpaces effects of disease severity in psoriasis vulgaris

**Figure 4:**
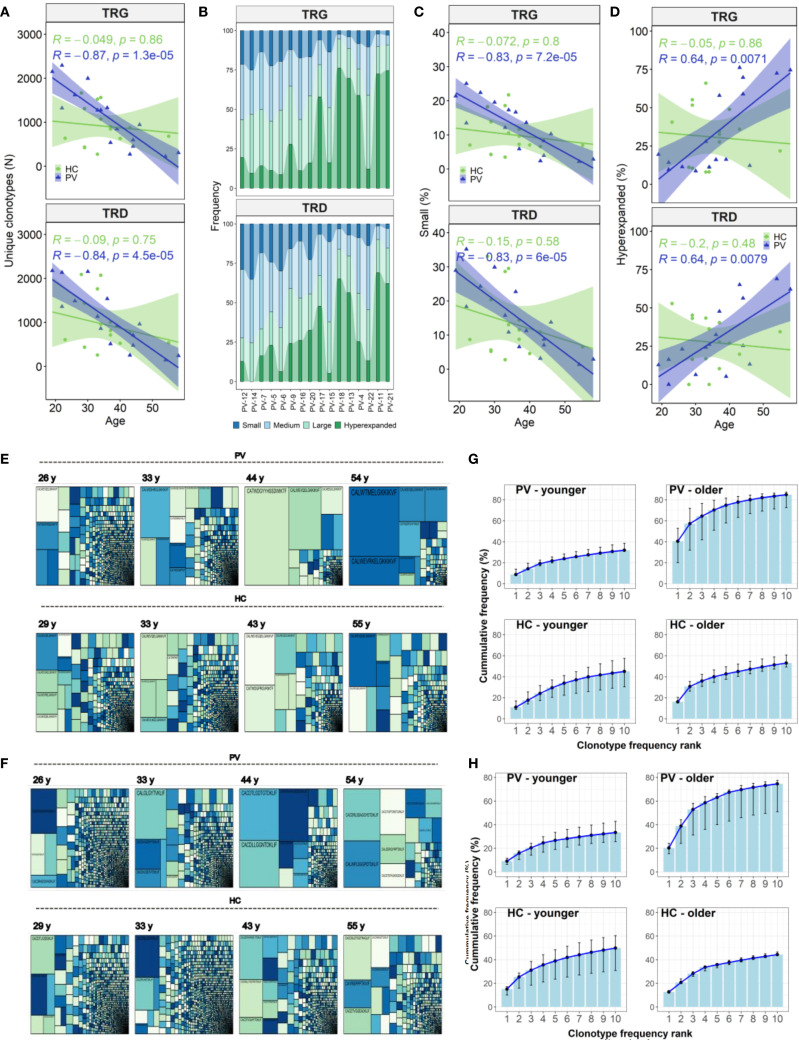
Age-related contraction of circulating γδ T cell repertoires in psoriasis vulgaris. **(A)** Advancing age in PV patients (N = 26) is associated with a decline in the total number of unique TRG and TRD clonotypes in peripheral blood, a trend not observed in healthy controls (N = 15). **(B)** Clonotype abundance stratified by frequency category – ‘small’ (≤0.05%), ‘medium’ (0.05–0.5%), ‘large’ (0.5–5%), and ‘hyperexpanded’ (>5%) – is visualized across PV patients ranked by age (x-axis). **(C, D)** The proportion of ‘small’ clonotypes within the TRG and TRD repertoires declines significantly with age in PV patients (**C**), while the proportion of ‘hyperexpanded’ clonotypes increases (**D**). These trends are not seen in healthy controls. **(E, F)** Treemaps comparing age-matched PV patients and healthy controls demonstrate increased clonal dominance in older PV patients within both TCRγ **(E)** and TCRδ **(F)** repertoires, relative to controls. **(G, H)** Cumulative frequency plots of the top 10 most abundant TRG **(G)** and TRD **(H)** clonotypes reveal greater clonal skewing in older PV patients (>40 years, N = 6), compared to younger PV patients (<40 years, N = 10) and age-matched healthy controls (N = 3 and N = 12, respectively). For each rank position (1–10), the median cumulative frequency and interquartile range (IQR) across individuals in each group were calculated. Spearman correlation coefficients (R) and corresponding p-values are indicated in panels **(A, C, D)**.

Owing to limited clonotype diversity in skin, we further focused on the γδTCR repertoire in peripheral blood, where age‐related effects paralleled, and often exceeded, those associated with PASI. In PV patients, advancing age correlated with dramatic reductions in total TCRγ and TCRδ (**Figure 4A**) clonotype counts, driven by loss of the ‘small’ and ‘medium’ TRG and TRD clonotype compartments (**Figure 4B, C**), and the expansion of the ‘hyperexpanded’ clones (**Figure 4B, D**). These shifts were most pronounced in TRGV9 and TRDV2 and extended across TRGV2–8 and TRDV3 subsets, where the loss of small clonotypes was coupled with declines in several diversity indices (**Supplementary Table 12**).

**Figure 5:**
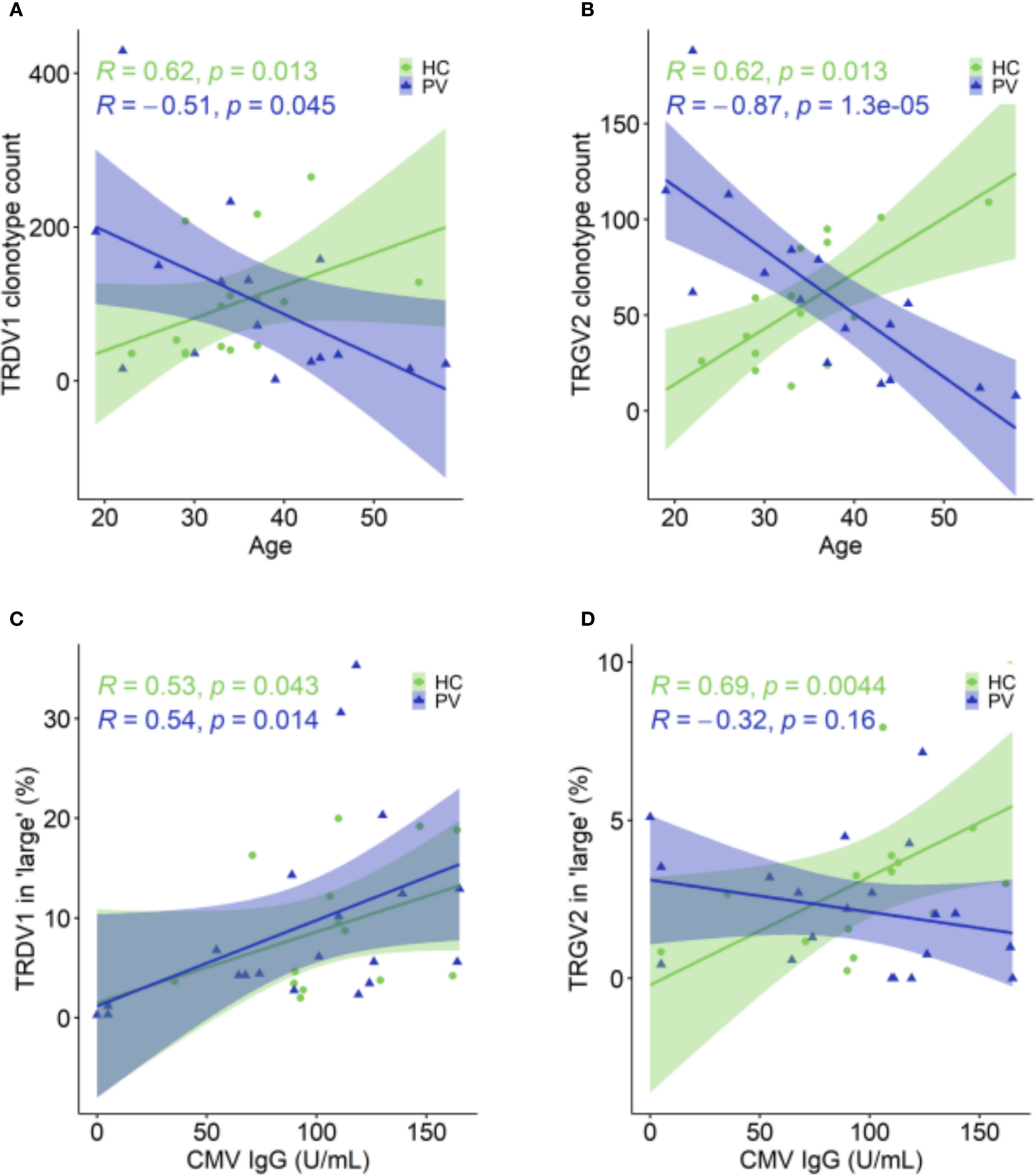
Age- and CMV-associated shifts in TRDV1 and TRGV2 clonotypes in healthy controls and psoriasis patients. **(A, B)** In HC (N = 15), TRDV1**(A)** and TRGV2 **(B)** clonotypes increase with age, whereas in PV patients (N = 26), these clonotype counts decline. **(C)** The proportion of TRDV1 clonotypes within the ‘large’ (0.5 – 5%) compartment positively correlates with CMV IgG levels in both PV (N = 30) and HC (N = 15). **(D)** The proportion of TRGV2 clonotypes in ‘large’ compartment is positively correlated with CMV IgG levels in HC, but this relationship is absent in PV patients. Spearman correlation coefficients (R) and p-values are shown for each group. In age-related correlation analyses panels **(A, B)**, four oldest PV donors were excluded due to the lack of matched healthy controls.

In healthy controls, these age-associated γδTCR repertoire constrictions were absent (**Figures 4E-H**). Instead, older healthy controls displayed expansions of TRDV1 and TRGV2 clonotype count (**Figures 5A, B**), especially within the large‐clone compartment (TRDV1: ρ = 0.527, P = 0.043, TRGV2: ρ = 0.643, P = 0.01), which correlated with CMV IgG titers (**Figures 5C, D**). In PV, CMV seropositivity further enriched TRDV1 in both ‘small’ (ρ = 0.553, P = 0.011) and ‘large’ clonotype compartments (**Figure 5C**), and deepened TRDV2 loss (ρ = -0.529, P = 0.017), highlighting a complex interplay of viral exposure, ageing, and chronic inflammation in PV. Importantly, flow cytometry confirmed that TRDV2 clonotype reductions reflected loss of clonotypic diversity rather than cell numbers, as blood Vδ2^+^ frequencies did not vary with PASI (ρ = –0.09, *P* = 0.74) or age (ρ = –0.36, *P* = 0.17). Moreover, higher CMV IgG titers in PV patients were linked to reduced Vδ2^+^ frequencies (ρ = –0.691, *P* = 7.38 x 10^-4^) and expansion of the Vδ1^-^Vδ2^-^subset (ρ = 0.541, *P* = 0.014) among total γδ T cells, supporting the contribution of CMV on γδTCR repertoire reshaping. However, CMV IgG levels in PV did not correlate significantly with age (ρ = 0.393, *P* = 0.086), disease duration (ρ = - 0.056, *P* = 0.815), or PASI (ρ = 0.198, *P* = 0.404), indicating that CMV is unlikely a primary driver of age- and disease severity-associated repertoire changes.

To pinpoint drivers of clonal expansion, we analyzed the top 10 clonotypes. Their cumulative frequencies increased with PASI (TCRγ: ρ = 0.542, P = 0.014; TCRδ: ρ = 0.457, P = 0.043) and age (TCRγ: ρ = 0.797, P = 2.2 × 10^-4^; TCRδ: ρ = 0.673, P = 0.004). Additionally, we summed the frequencies of the first 10 clonotypes within distinct TRDV and TRGV gene segments, independent of their position in the overall repertoire ranking. This revealed segment-specific associations: TRDV3 frequencies correlated with disease severity (ρ = 0.517, P = 0.023), TRDV2 with age (ρ = 0.551, P = 0.027), and TRGV4 with disease duration (ρ = 0.470, P = 0.037). Stratification by age (< 40 vs. ≥ 40 years) and PASI (≤ 10 vs. > 10) further showed that older patients, but not those with higher PASI, harbor significantly higher cumulative frequencies of top clonotypes (TCRγ: 83.4% vs. 30.7%, P = 0.008; TCRδ: 73.2% vs. 32.3%, P = 0.026). Patient age also correlated with longer CDR3δ lengths (ρ = 0.601, P = 0.014) and increased NDN additions in non-TRDV2 clonotypes (ρ = 0.613, P = 0.034), with females exhibiting longer CDR3δ2 regions than males (48.1 [46.3 – 49.8] vs 52 [50.8 – 52.9], P = 0.006; M vs F), patterns absent in controls. In summary, aging in PV patients triggers a pronounced contraction of peripheral γδTCR diversity alongside expansion of dominant clones, surpassing changes driven by disease severity. In contrast, healthy individuals exhibit CMV-associated repertoire expansions with age, whereas in PV the interplay of aging, CMV exposure, and chronic inflammation predominantly narrows γδTCR diversity without altering cell numbers.

### 3.6 Peripheral γδTCR repertoires in psoriasis show polyclonal diversification and partial blood-skin sharing

**Figure 6:**
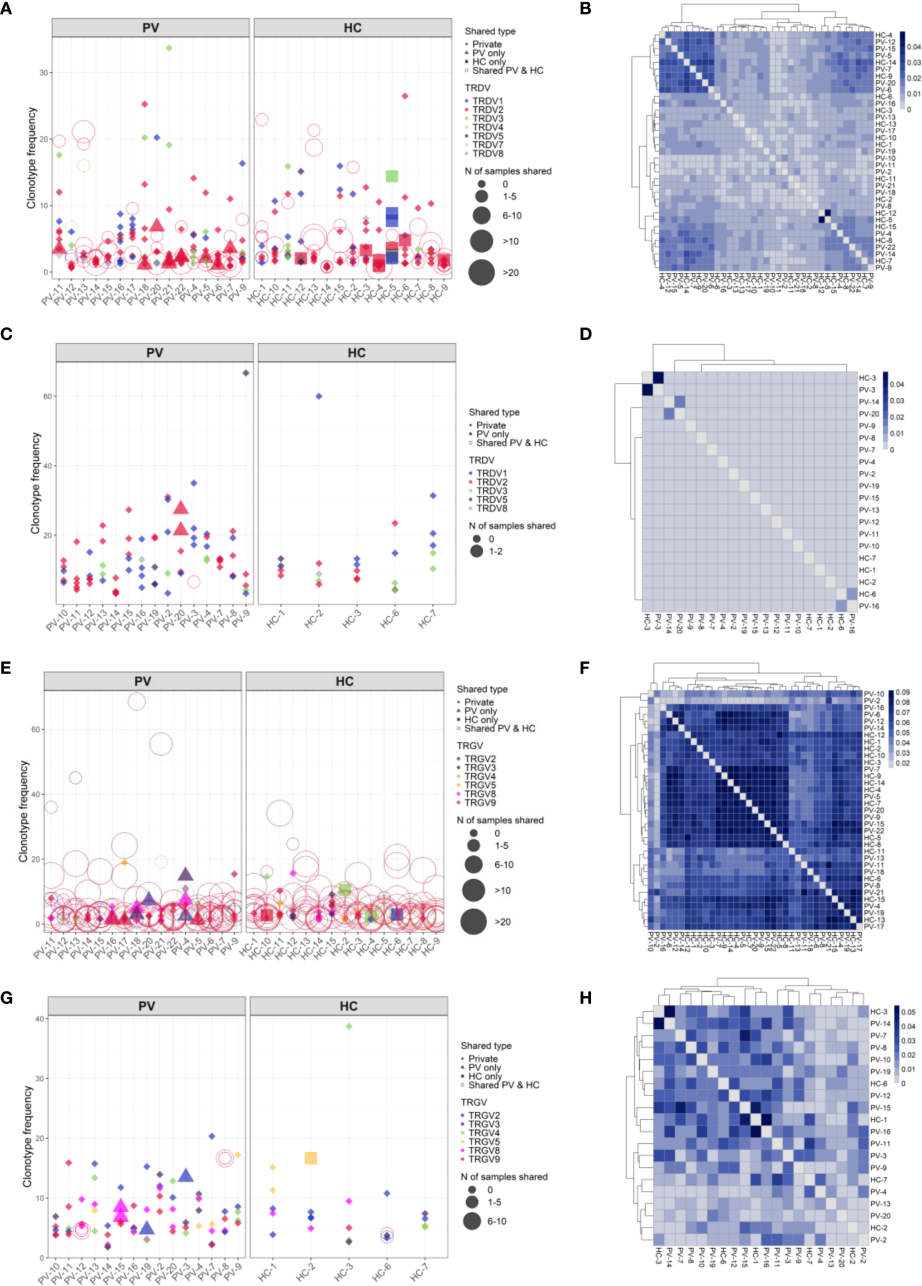
TCRδ clonotypes are predominantly private in both blood and skin, whereas TCRγ repertoires display greater inter-individual sharing. Frequencies of the top TCRδ and TCRγ clonotypes in peripheral blood and skin of psoriasis vulgaris (PV) patients and healthy controls (HC). Top 10 clonotypes are shown unless fewer were detected; in skin samples with low TCRδ clonotype counts, the top 5 are shown. Each point represents a unique clonotype; symbol size indicates the number of individuals sharing that clonotype, and color denotes the V gene segment used. TCRδ clonotypes are largely private in both blood **(A)** and skin **(C)**. In contrast, TCRγ repertoires demonstrate higher public sharing particularly in blood **(E)**, while skin-derived TRG clonotypes **(G)** are more private but still more commonly shared than their δ-chain counterparts. **(B, D, F, H)** Pairwise Jaccard similarity matrices show the extent of inter-individual clonotype overlap. TCRδ repertoires in blood **(B)** and skin **(D)** display low similarity between individuals, whereas TCRγ repertoires exhibit higher overlap in blood **(F)** and moderate overlap in skin **(H)**, consistent with greater public repertoire characteristics.

**Figure 7:**
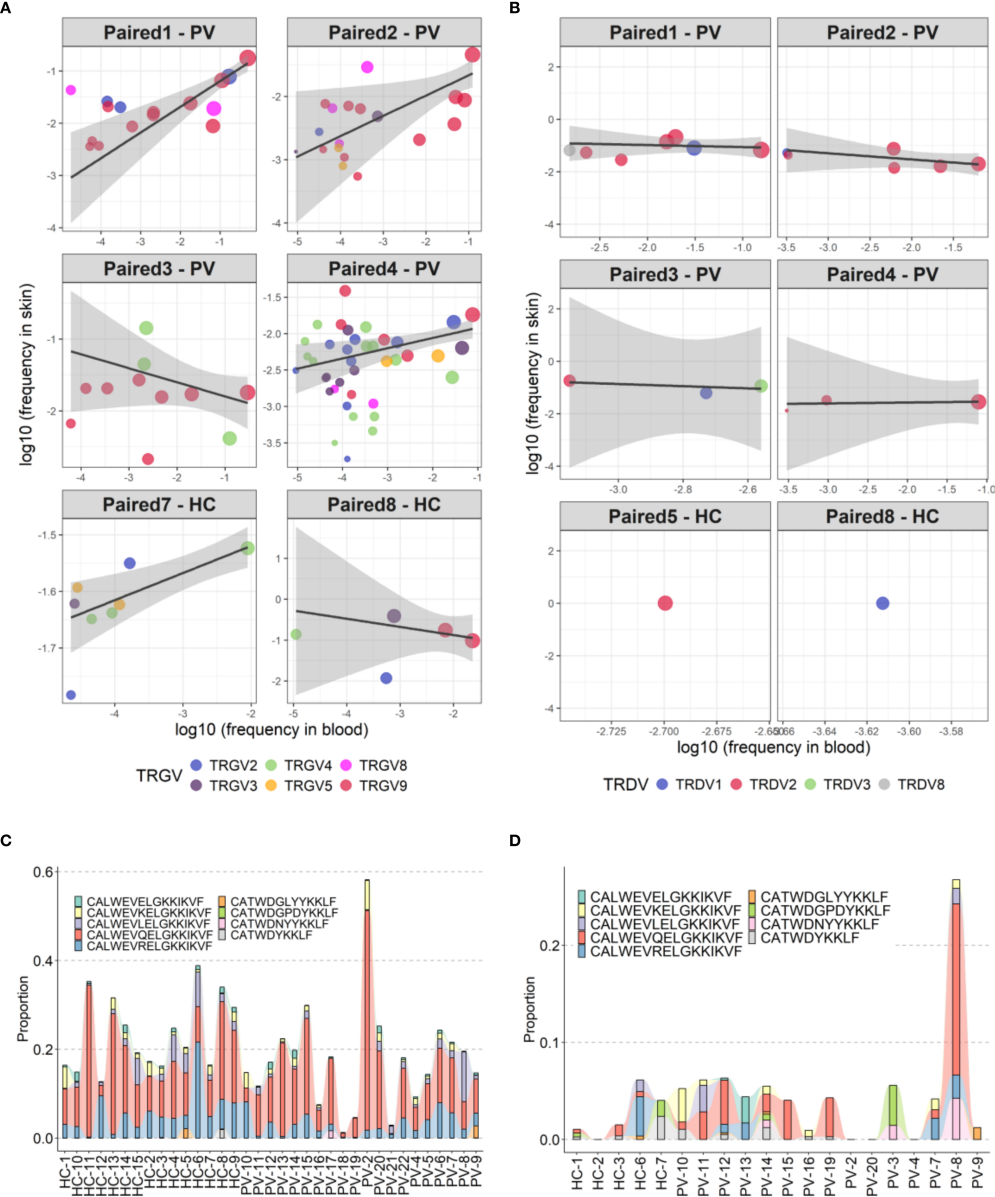
Partial overlap of circulating and skin-resident TCRγδ clonotypes in psoriasis and healthy controls. **(A, B)** Clonotype sharing between peripheral blood and lesional skin for TCRγ **(A)** and TCRδ **(B)** repertoires in six individuals. Each point represents a unique clonotype; point size reflects the mean frequency across compartments (log-transformed), and color indicates the TRGV gene segment. Trend lines depict weighted linear models, emphasizing relative distribution without formal regression statistics, which were omitted due to low clonotype counts in some samples. **(C, D)** Frequency distribution of nine public TCRγ clonotypes shared across peripheral and skin-resident γδ T cells in psoriasis patients and healthy controls, shown separately for peripheral blood **(C)** and skin **(D)**.

Analysis of paired peripheral and cutaneous γδTCR repertoires revealed extensive diversity but limited disease-specific clonotypes in psoriasis. In blood, 27,073 TCRδ and 15,364 TCRγ clonotypes were identified (**Supplementary Table 5**), with markedly lower counts in skin (356 TCRδ; 1,215 TCRγ). Most TCRδ clonotypes were private (7.5% public in blood; 0.84% in skin; **Figures 6A-D**), whereas TCRγ repertoires were more public (18.8% and 10.29%, respectively; **Figures 6E-H**). Within the public blood compartment, TRDV2 dominated TCRδ and TRGV9 dominated TCRγ usage, with lower contributions from TRDV1/3 and TRGV2/3/4/5/8 (**Supplementary Table 13**). Although several clonotypes were enriched in PV or healthy donors, none reached statistical significance after Fisher’s exact test with Benjamini-Hochberg correction. Jaccard index (JI) analysis showed reduced intra-PV overlap for TRGV8 (PV–HC overlap 0.009 [0–0.016], P = 0.002; **Supplementary Figure 7A**) and a similar but less pronounced pattern for TRGV4 (**Supplementary Figure 7B**), together with elevated D50 values, indicating broader, polyclonal diversification in psoriasis blood. In contrast, skin TCRγ overlap and JI values were similar between PV and HC, with only ten public skin TCRγ clonotypes absent from blood (**Supplementary Table 14**). Paired blood-skin analysis in eight participants (5 PV, 3 HC) further revealed sharing of γδTCR clonotypes in six participants (4 PV, 2 HC; **Figures 7A-B**). This involved nine public clonotypes, including five TRGV9-TRGJP rearrangements such as germline-encoded CALWEVQELGKKIKVF, CALWEVRELGKKIKVF, and CALWEVLELGKKIKVF, three TRGV4-TRGJ2 and one TRGV2-TRGJ2 variant (**Figures 7C-D**). Four of these nine occurred exclusively in lesional skin. Limited but mostly private TCRδ overlaps were also detected, mainly TRDV2–TRDD3–TRDJ1 rearrangements, including two public clonotypes (CACDVLGDPYTDKLIF and CACDRLGDTDKLIF) that were more frequent in PV blood (16 PV vs. 8 HC and 4 PV vs. 1 HC, respectively). Comparison with the Harden et al. dataset (36), confirmed detection of all nine public blood-skin TCRγ clonotypes in lesional skin, with several absent from healthy skin. Notably, CALWEVRELGKKIKVF, enriched in lesional but not healthy skin, also matched lesional pool from Harden et al., supporting recruitment of circulating Vγ9Vδ2 T cells (23) to inflamed tissue.Together, these findings point to systemic polyclonal γδ diversification in psoriasis with stable cutaneous repertoires and partial blood–skin sharing of public clonotypes consistent with infiltration of circulating γδT cells.

### 3.7 Transcriptional rewiring of circulating γδ T cells in psoriasis reveals a hyperactivated, cytotoxic, tissue-homing effector state

**Figure 8:**
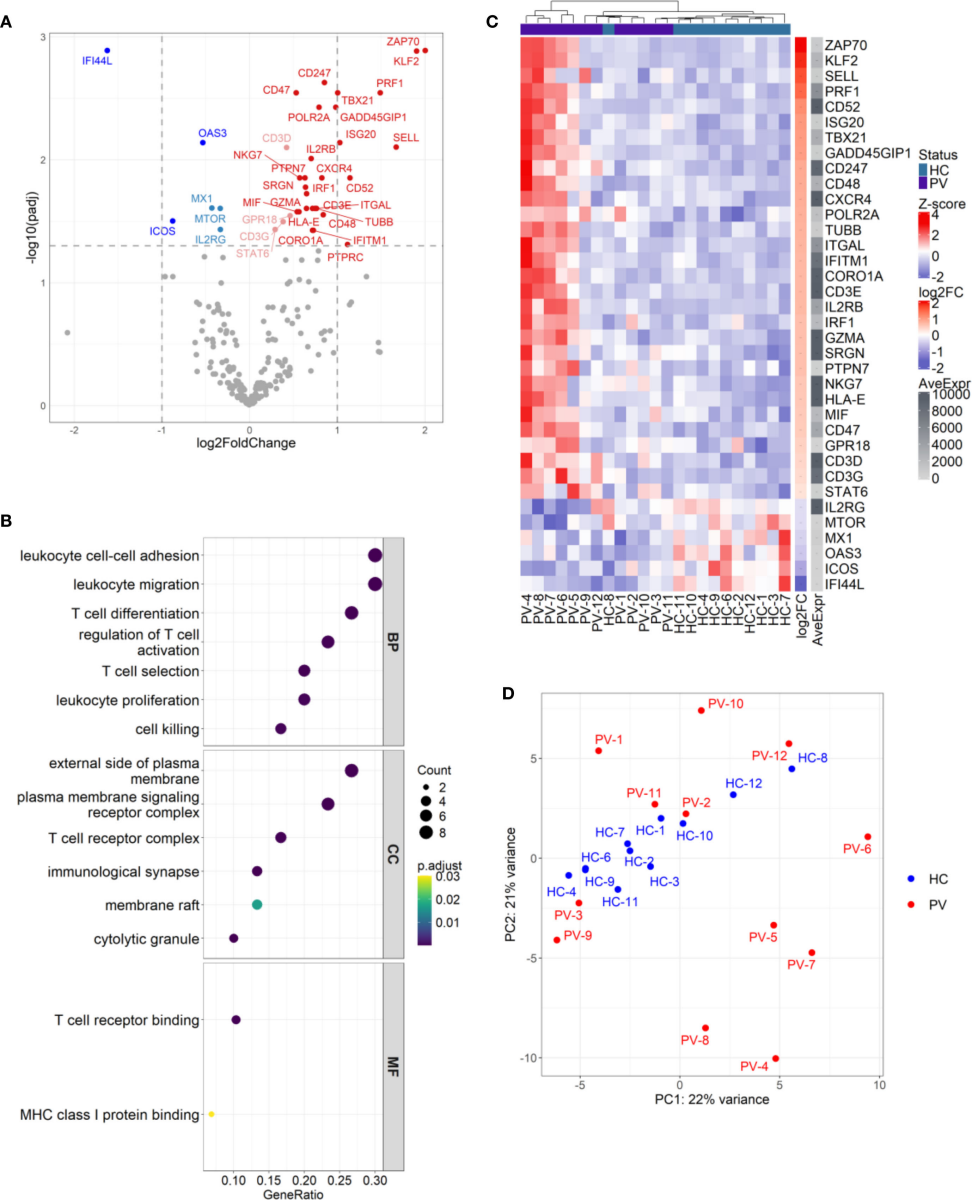
Circulating γδ T cells in psoriasis patients display differential effector gene signatures with inter-individual heterogeneity. **(A)** Differential expression analysis of 300 genes in γδ T cells from psoriasis patients (N = 12) versus healthy controls (N = 11) revealed 36 significantly modulated genes (30 upregulated, 6 downregulated; adj.p < 0.05). Color coding reflects significance and fold change: dark red/blue for log_2_FC > ± 1, light red/blue for log_2_FC < ± 1; non-significant genes are grey. **(B)** Gene ontology (GO) enrichment analysis of upregulated genes identified pathways involved in T cell activation, adhesion, migration, and cytotoxicity. Dot size represents gene ratio; color indicates adjusted p-value. GO terms are grouped by BP (Biological Process), CC (Cellular Component), and MF (Molecular Function). **(C)** Heatmap of differentially expressed genes (rows) across individual samples (columns), with Z-score normalization. Samples are hierarchically clustered, and group identity is indicated by a color bar. **(D)** Principal component analysis (PCA) plot shows partial segregation of a subset of psoriasis samples (PV-4 to PV-9) from other patients and controls, indicating inter-individual transcriptional heterogeneity.

To probe the functional alterations of circulating γδ T cells in psoriasis, we analyzed gene expression of 395 immunologically relevant genes in FACS-sorted cells from 24 participants (12 PV, 12 HC). Differential expression analysis revealed 36 genes with altered expression (30 upregulated, 6 downregulated), describing a transcriptional profile that positions circulating γδ T cells as active participants in the inflammatory milieu of psoriasis (**Figure 8A**, **Supplementary Table 15**).

Gene ontology and pathway enrichment analysis highlighted coordinated upregulation of processes orchestrating T cell activation, selection, differentiation, adhesion, migration, and cytotoxic function, particularly at immune signaling sites like the TCR complex, immunological synapse, and cytolytic granules (**Figure 8B**). Consistent with heightened activation, TCR signaling components (*CD3D/E/G, CD247*, and *ZAP70*) and the co-stimulatory *CD48* (41) were upregulated, with enrichment in the KEGG “T cell receptor signaling pathway” (adj. p value: 6.34 x 10^-4^) and the Biocarta TCR pathway (adj. p value: 4.93 x 10^-6^; **Supplementary Figure 8**). A robust type-1 effector phenotype was supported by upregulation of *TBX21* and *IRF1*, IFN-γ-inducible genes (*IFITM1*, *ISG20*) and the stress-responsive, IFN-γ promoting gene *GADD45G* (42). Elevated *IL2RB*, implied increased sensitivity to IL-2 and IL-15 cytokines that potentiate γδ T cell cytotoxicity by inducing perforin, T-bet, and Emes transcription (43). Correspondingly, cytotoxic mediators such as *PRF1*, *GZMA*, *SRGN*, and *NKG7* were upregulated, paralleling signatures seen in αβ T cells in psoriasis (44–46). Enrichment of the KEGG “Natural killer cell mediated cytotoxicity” pathway (Gene ratio: 6/20, adjusted p value: 2.53 x 10^-4^) and the Biocarta cytotoxic T lymphocyte (CTL) pathway (Gene ratio: 6/14, adjusted p value: 8.3 x 10^-9^) (**Supplementary Figure 8** and **Supplementary Figure 9**) further supported this cytotoxic imprint. Upregulation of *KLF2*, *SELL*, and *ITGAL* indicated increased trafficking capacity (47, 48), while elevated *CXCR4* expression suggested directed migration to skin, consistent with reports of CXCR4 upregulation in psoriatic lesions (44, 49), and dermal γδ T cells (6). Expression of *CD47* and *HLA-E* implied immune evasion potential, via resistance to phagocytosis (50) and engagement with inhibitory NK receptors (CD94/NKG2A) (51). In contrast, antiviral genes *MX1*, *OAS3*, and *IFI44L* were suppressed in γδ T cells in psoriasis, suggesting that these cells may be less primed to combat viral threats; alternatively, this finding could reflect an upregulation of these genes within the control group. Importantly, peripheral γδ T cell subset proportions (Vδ1, Vδ2, Vδ1^-^Vδ2^-^) did not differ between groups (**Supplementary Table 16**), confirming these transcriptional shifts were not due to compositional bias. Adding a further layer of complexity, principal component analysis (PCA) revealed a heterogeneity in the upregulated gene signature across psoriasis patients, with a subset of individuals (PV-A: PV-4, PV-5, PV-6, PV-7, PV-8, and PV-9) clustering apart from both controls and other PV patients (termed PV-B), who interspersed with healthy controls (**Figures 8C, D**). Comparing gene expression between different subsets (PV-A vs. HC, PV-B vs. HC, and PV-A vs. PV-B) showed that the psoriasis-associated γδ T cell signature was largely driven by the PV-A subgroup. In the PV-A vs. HC comparison, all previously identified upregulated genes remained, along with additional elevated, as well as downregulated transcripts (**Supplementary Figure 10**). Of note, the statistical significance of certain genes, such as *ZAP70*, was markedly enhanced (adjusted p-value dropped from 0.001 to 3.78 x10^-23^, and log_2_fold increased from 2 to 3.01). In contrast, the PV-B vs. HC comparison revealed no significant DEGs, supporting the idea that PV-B patients have a transcriptional profile akin to healthy individuals. However, while many of the upregulated genes in the PV-A vs. HC analysis were also present in the PV-A vs. PV-B comparison, several transcripts (*SELL*, *CD247*, *POLR2A*, *SRGN*, *NKG7*, *CD3D*, *CD3G*, and *STAT6*) were not differentially expressed in the PV-A vs. PV-B comparison, provoking the thought that two subgroups represent different levels along a continuum of immune activation, with PV-A likely reflecting a more robust activation, and PV-B indicating a subtler, intermediate level of response. Interestingly, this heterogeneity could not be explained by age, PASI score, disease duration, sex, CMV status, and γδ T cell subset proportions, leaving the underlying drivers of this variability unresolved. This divergence may reflect distinct disease endotypes, differences in immune microenvironments, or other yet unidentified factors shaping γδ T cell function in psoriasis.

## 4 Discussion

Psoriasis vulgaris (PV) is a chronic inflammatory dermatosis where prominent role has been assigned to T cells. Most prior studies in humans and mice, have focused on αβ T cells, leaving the roles of γδ T lymphocytes relatively understudied. In this study, we combined flow cytometry, mRNA profiling and TCR sequencing for integrated analysis of circulating and cutaneous γδ T cells of PV patients and healthy controls, correlating these data with clinical, demographic, and anthropometric features. We found that psoriasis induces distinct and tissue-specific remodeling of the γδ T cell compartment, including alterations in cell frequency, gene expression, and TCR repertoire architecture. These changes were shaped by a complex interplay of sex, age, disease severity and chronicity, and differed between blood and lesional skin of PV patients.

Consistent with previous studies (6, 52–54), psoriatic lesions were enriched for CD3^+^ T cells, while total γδ T cell frequencies remained comparable to healthy skin. Nonetheless, TRG/TRD transcripts positively correlated with disease severity and duration, suggesting increased transcriptional activity or clonal skewing rather than true cellular expansion. An age-associated decline in cutaneous γδ T cells was observed in psoriasis but not in healthy skin, suggesting that chronic inflammation perturbs local γδ T cell homeostasis typically maintained during physiological ageing (55). Contrary to earlier reports of Vγ9Vδ2 enrichment in psoriatic lesions (8, 19), we observed no expansion of the Vδ2^+^ compartment. Because our panel lacked Vγ9-chain staining, the Vδ2^+^ population likely included non–Vγ9 tissue-resident cells (23, 56), preventing direct assessment of Vγ9Vδ2 dynamics and potentially masking previously reported subset-specific alterations (8, 19). Instead, we observed enrichment of Vδ1^-^Vδ2^-^ subset in lesional skin, hinting at expansion of unconventional or tissue-adapted subsets. Interestingly, skin Vδ2^+^ frequencies increased with higher PASI scores in female patients only, suggesting potential sex-specific contribution to PV immunopathology. This sex-specific pattern extended to the peripheral blood, where male patients showed reduced Vδ2^+^ cells, consistent with prior reports (8, 19), though not previously associated with sex. Whether this pattern reflects true biological variation or is influenced by cohort composition remains to be clarified. Of note, healthy female controls in our cohort displayed lower baseline γδ and Vδ2^+^ frequencies than males, contrasting prior reports (30, 31), which may have attenuated case-control differences in our unstratified analyses. Importantly, Laggner et al. demonstrated that circulating Vγ9Vδ2 reductions were largely restricted to the skin-homing CLA^+^ subset (8) which we did not assess, potentially explaining differences between our findings and previous reports. In addition to numerical differences, female patients displayed a higher prevalence of longer TRDV2-TRDJ3 rearrangements, diverging from the canonical TRDV2-TRDJ1 usage typically seen in healthy individuals (23). These structural differences were absent in healthy male and female controls, implicating psoriasis as the primary driver of this repertoire divergence. Longer TRDV2-TRDJ3 rearrangements have been previously associated with extrathymic maturation of fetal γδ T cells (57), but their role or origin in the peripheral repertoire of adult female psoriasis patients remains unclear. However, given the male-biased composition of our cohort, these observations warrant validation in larger, sex-balanced populations to clarify their relevance to disease pathogenesis.

In addition to sex-associated differences, psoriasis was associated with marked structural changes of peripheral and cutaneous γδTCR repertoires. As an initial observation, both healthy and lesional skin exhibited shorter TRG CDR3 regions compared to blood, largely due to alternative TRGV-J gene pairing. This was accompanied by a bimodal distribution of TRGV9 CDR3 lengths, driven by increased usage of TRGV9-TRGJ2 combinations, and distinct TRD CDR3 length profiles between blood and skin. In blood, TRDV2 distributions were predominantly Gaussian-like, while TRDV1 and TRDV3 exhibited bimodal profiles. In contrast, the skin repertoire showed a more balanced TRDV1/TRDV2 representation with a bimodal and elongated TRDV2 landscape, and only a few dominant TRDV1 and TRDV3 peaks, suggesting selective pressures and receptor skewing in the cutaneous microenvironment. Psoriasis-specific repertoire remodeling was apparent beyond these tissue-specific features. Psoriasis severity correlated with increased CDR3 length and junctional diversity, particularly within TRGV8 and TRDV2 clonotypes. Additionally, longer TRDV1/2/3 rearrangements were largely absent in healthy skin, supporting a model in which inflammatory signaling in lesional tissue drives enhanced receptor remodeling and clonal diversification – consistent with findings in rheumatoid arthritis, where longer TRD rearrangements were similarly enriched (58). These structural shifts mirror those described in peripheral γδ T cells upon aging (32, 59). Specifically, naive subsets in young adults typically exhibit Gaussian-like TRD CDR3 distributions, whereas central memory (T_CM_ CD27^+^CD45RA^-^) and effector memory (T_EM_ CD27^-^CD45RA^-^) subsets gradually adopt more skewed distributions, and terminally differentiated effector memory cells (T_EMRA_ CD27^-^CD45RA^+^) display dominant peaks and reduced diversity. Supporting this interpretation, Vγ9Vδ2 cells in psoriasis patients have previously been shown to adopt a CD27^-^CD45RA^+^ terminal phenotype, accompanied by contraction of the central memory pool (19). Whether similar subset redistribution occurs within lesional skin, and whether this contributes to the distinct repertoire features we identified here, remains to be clarified in future studies.The most striking hallmark of psoriasis-associated γδTCR dysregulation was a marked attrition of peripheral repertoire diversity coupled with selective depletion of clonotypes in the skin. Although total clonotype counts were comparable between groups, higher PASI scores were associated with lower repertoire diversity, loss of rare clonotypes, and expansion of dominant clones, particularly within the TRGV9 and TRDV2 subsets. This loss of peripheral diversity was consistent across multiple analytical metrics and coincided with depletion of hyperexpanded TRGV8 clones in lesional skin. At the same time, medium-sized TRGV4 clonotypes became increasingly represented in both blood and skin, suggesting targeted remodeling of circulating and tissue-resident γδ subsets in PV. A particularly notable finding was the loss of shared/public TRGV8, and to a lesser extent, TRGV4 clonotypes in PV blood, indicating increasing peripheral repertoire heterogeneity. This divergence was, moreover, associated with elevated TRGV8 and TRGV4 D50 values in the blood of psoriasis patients, consistent with polyclonal expansion and diversification rather than dominance of few expanded clones. These features parallel findings in γδ and αβ T cells in autoimmune (58) and chronic inflammatory conditions (60), where persistent antigenic stimulation drives personalised, heterogeneous immune responses. By contrast, healthy individuals showed more conserved, public TRGV4 and TRGV8 repertoires, consistent with CMV-associated clonal expansions of Vγ8 and Vγ4 TCRs (61), highlighting the disease-specific nature of the repertoire changes observed in PV.

Beyond disease severity, aging also emerged as a dominant, independent driver of the peripheral γδTCR repertoire contraction, often exceeding the effects of disease severity. In blood of PV patients, increasing age correlated with a progressive loss of small and medium-sized clonotypes, particularly within TRGV9 and TRDV2 subsets, along with compensatory rise in hyperexpanded clones, suggestive of repertoire contraction and skewing. While healthy individuals displayed age-related expansions in TRGV2 and TRDV1 clonotypes, likely linked to CMV seropositivity (26, 35, 62–64), in PV, CMV further intensified TRDV2 depletion and TRDV1 expansion. However, as CMV titers did not correlate with age or disease severity in PV, CMV alone could not fully account for the age and disease-related repertoire changes. Importantly, this collapse in peripheral TRDV2 repertoire in PV occurred independently of changes in Vδ2^+^ cell frequencies, pointing to true repertoire contraction rather than a reduction in circulating cell numbers. Aging was also associated with higher cumulative frequencies of dominant clones and elongation of CDR3δ regions, primarily due to increased NDN insertions, particularly in non-TRDV2 rearrangements. These age-associated patterns resemble repertoire changes described in both γδ (65) and αβ T cells (66, 67) of older individuals, where longer CDR3 regions contribute to enhanced cross-reactivity and reduced TCR specificity (68).The observed contraction in TRGV9 and TRDV2 repertoires with increasing age and disease severity resembles the features of “late-stage differentiated” CD27^-^CD28^-^ CD16^+^ Vγ9Vδ2 cells (69). Specifically, CD27^-^ Vγ9Vδ2 T subsets show significantly fewer clonotypes and lower diversity in their TCRδ repertoires compared to their CD27^+^ (CD27^+^CD28^+^) counterparts (23, 69), suggesting that more severe or long-standing disease may be associated with a skewing toward cytotoxic, effector-like γδ T cells. Supporting this phenotypic transition, transcriptomic profiling of circulating γδ T cells in our cohort revealed a strongly pro-inflammatory and cytotoxic signature, marked by elevated TCR signaling (*CD3D/E/G*, *ZAP70*, *CD247*), cytotoxic effectors (*PRF1*, *GZMA*, *NKG7*, *SRGN*), Th1 polarization (*IL2RB*, *TBX21*, *IRF1*, *GADD45G*), and tissue-homing markers (*CXCR4*, *KLF2*). These transcriptional features are consistent with functionally primed cells capable of migrating to inflamed tissue (47, 70, 71) and contributing to psoriatic pathology (43, 46, 49, 72). Notably, they overlap with the transcriptional profiles of NK and CD8^+^ T_EM_/T_TEMRA_ αβ T cells in psoriasis (44–46, 72), positioning γδ T cells as integral components of the effector immune response in this disease. However, substantial inter-individual variability was observed, with some patients showing transcriptional profiles similar to healthy controls. These findings align with prior reports of cellular and molecular heterogeneity in psoriasis (72–74) and imply the existence of distinct disease endotypes that may vary with severity and duration, arguing in favor of a longitudinal studies integrating single-cell data to uncover these dynamics and their clinical implications.

Several public TRGV9-TRGJP clonotypes, including germline-encoded variants, were consistently observed in blood and lesional skin and also detected in external psoriasis datasets (36), indicating their potential recurrence in inflamed tissues. However, their frequent presence in healthy individuals, as shown for CALWEVQELGKKIKVF and CALWEVRELGKKIKVF, in both our data and prior studies (23, 36, 75), suggests that they are widely distributed, skin-homing variants with limited association to psoriasis. Aside from these ubiquitously shared variants, most clonotypes remained private, particularly in the skin-derived TCRδ repertoires, while TCRγ clonotypes were more commonly shared across individuals and compartments. Nonetheless, nearly half of all skin-derived TCRγ (41.07%) and a smaller fraction of TCRδ (17.42%) clonotypes were also present in blood, similarly distributed between PV and controls, indicating that cutaneous γδ T cells may derive from circulating pools in both health and disease. Of interest, few public clonotypes appeared restricted to either PV or healthy blood samples, but none showed statistically significant enrichment after correction. Similarly, skin-specific clonotypes, such as CALWEVKELGKKIKVF in PV lesional skin, showed intriguing but statistically inconclusive patterns. Several public lesional skin clonotypes also overlapped with those reported by Harden et al. (36), further hinting at possible disease relevance. However, the lack of statistical significance and the small sample size preclude firm conclusions regarding their functional roles or diagnostic utility.Future application of single-cell TCR sequencing could allow unambiguous linking of γ- and δ-chains within individual cells and greatly improve the interpretability of our repertoire data.

In summary, psoriasis drives compartment-specific, inflammation-associated changes of the γδTCR repertoire, marked by peripheral contraction, cutaneous reshaping, and structural instability influenced by age, disease, and sex. A central novelty of our study is the comprehensive characterisation of the diversity and frequency of multiple γδ T cell subsets, including, but not limited to, Vγ9Vδ2 in both blood and healthy/lesional skin. By capturing a broader fraction of the γδ-cell pool, our work extends current understanding and may ultimately facilitate the development of more accessible algorithms for routine monitoring of disease activity. Although robust evidence for public disease-specific clonotypes remain elusive, gene expression profiling identifies a functionally active, tissue-homing subset of γδ T cells likely to contribute to pathogenesis in a patient-specific manner. Collectively, these findings advance our understanding of PV by linking repertoire architecture and functional states to disease heterogeneity, providing a framework for stratifying patients based on immune profiles. Furthermore, γδ T cells emerge as promising targets for therapeutic intervention, either by modulating their recruitment, effector functions, or clonal composition. Future studies employing larger, sex-balanced cohorts, single-cell TCR/RNA sequencing, and longitudinal sampling are warranted to refine these observations and translate them into clinical strategies.

## Data Availability

The RNA-seq and TCR-seq datasets from peripheral blood samples can be found in the NCBI SRA, under the BioProjects PRJNA1293314 (https://www.ncbi.nlm.nih.gov/sra/PRJNA1293314) and PRJNA1292788 (https://www.ncbi.nlm.nih.gov/sra/PRJNA1292788), respectively. TCR-seq data from skin samples will be made available under BioProject PRJNA1293805 upon publication of forthcoming analysis focused on αβTCR repertoires.
