# Supplementary material for: Psoriasis vulgaris leaves a dynamic imprint on circulating and skin γδ TCR repertoires shaped by disease severity, age, and sex
Source: Front Immunol. 2025 Oct 17;16:1670364. doi: 10.3389/fimmu.2025.1670364 (PMC12576707; doi:10.3389/fimmu.2025.1670364)
Supplement: Supplementary file 1 [file DataSheet1.pdf]

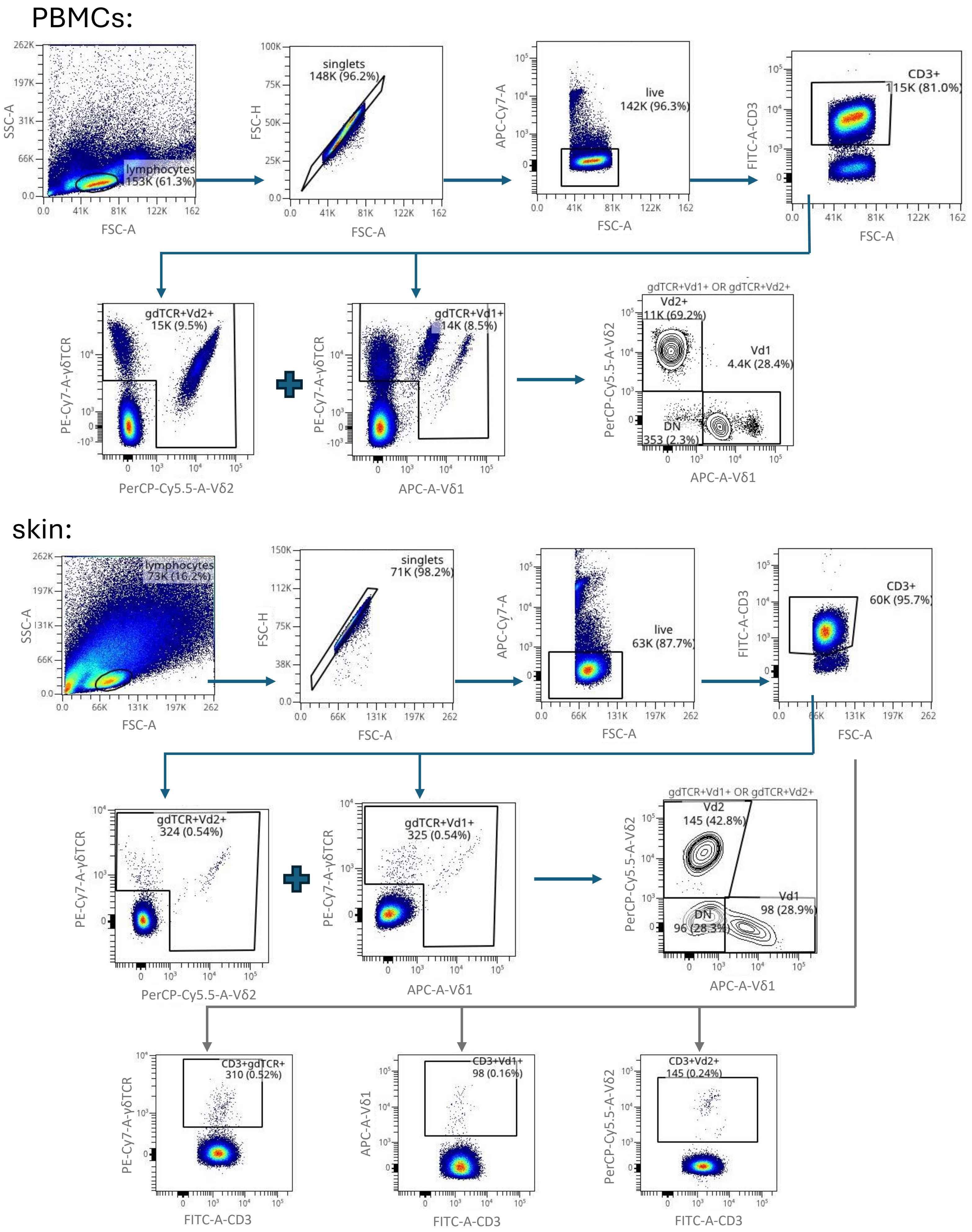

Supplementary Figure 1. **Gateing strategy for circulating and skin γδ T cell populations.** *Vδ1$^+$, Vδ2$^+$, and double-negative; DN (Vδ1$^-$Vδ2$^-$) γδ T cells were gated from singlet, live, CD3$^+$ T cells using a Boolean gating strategy: γδT$^+$Vδ1$^+$ OR γδT$^+$Vδ2$^+$. This approach compensates for the incomplete detection of γδ T cells by the pan-γδTCR antibody, as shown in the left-lowest plot, where total γδ T cell numbers are underestimated compared to the Boolean γδT$^+$Vδ1$^+$ OR γδT$^+$Vδ2$^+$ strategy. Vδ1$^+$ and Vδ2$^+$ cell counts from the Boolean gate match those obtained when gating directly from the CD3$^+$ population.*

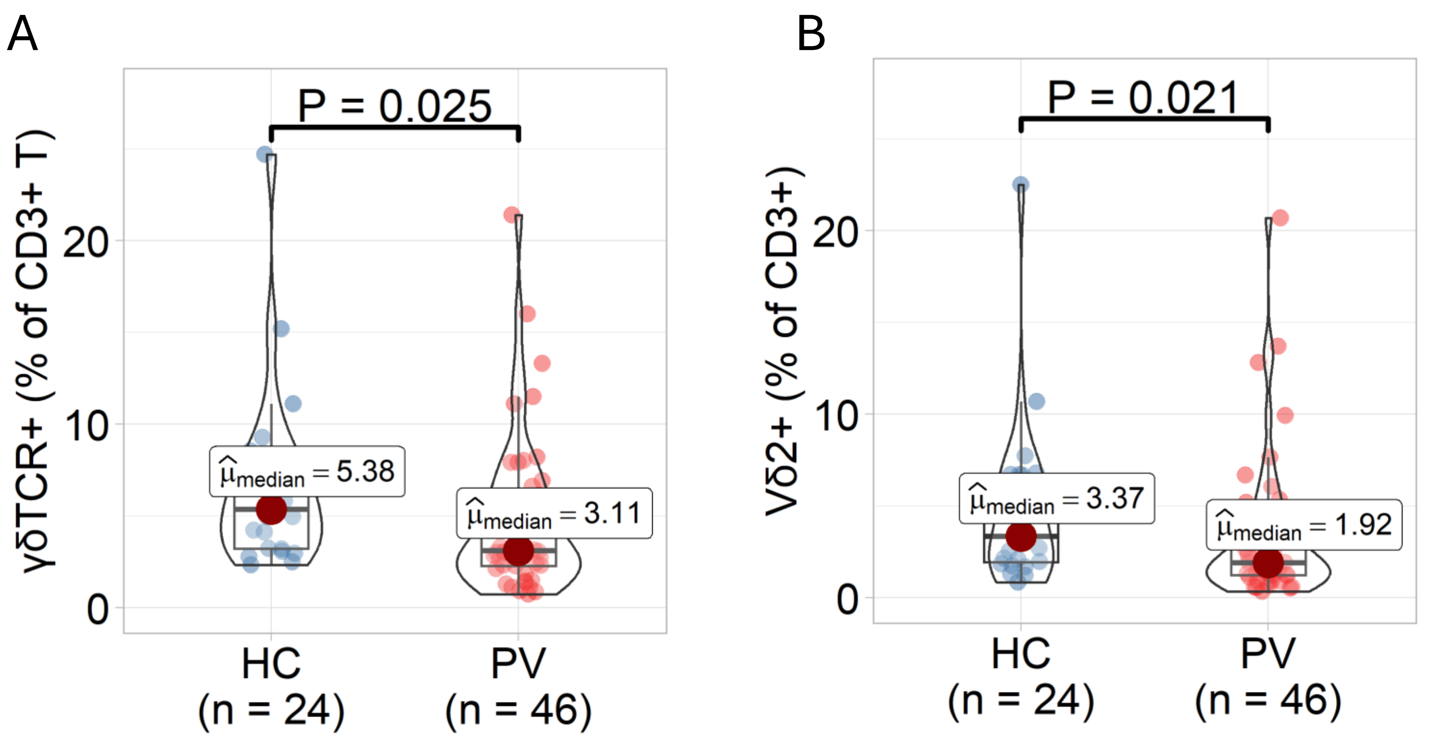

Supplementary Figure 2. Reduced circulating γδ T and Vδ2 cell frequencies in male psoriasis patients compared to healthy men. *Proportions of A) total γδ TCR+ and B) Vδ2+ cells within CD3+ T cells are significantly lower in male psoriasis patients (PV) compared to healthy male controls (HC). P-value calculated by Mann-Whitney U test.*

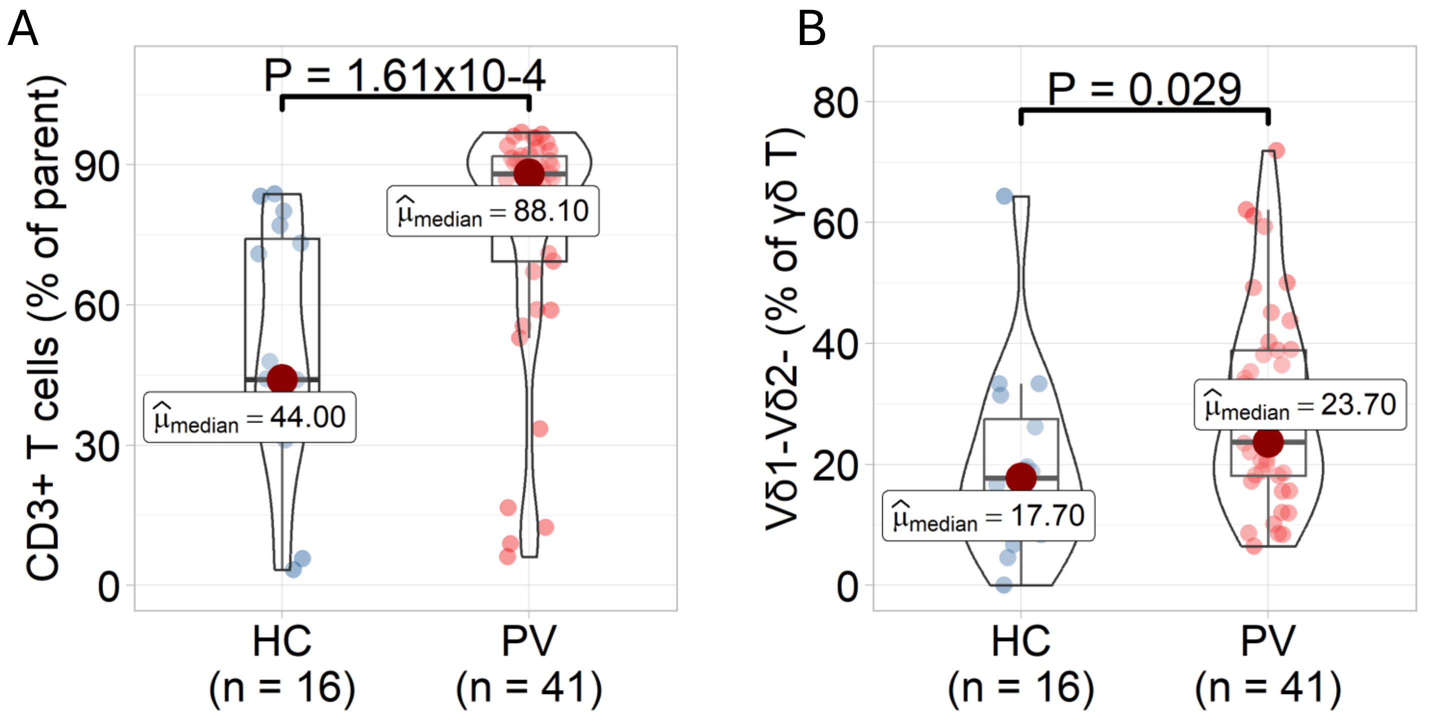

Supplementary Figure 3. Increased proportions of CD3+ and Vδ1-Vδ2- cells in psoriatic lesions. *Proportions of A) CD3+ cells within parent (live singlet lymhphocytes) population and B) Vδ1-Vδ2- cells within γδ T cells are significantly higher in psoriasis patients (PV) compared to healthy controls (HC). P-value calculated by Mann-Whitney U test.*

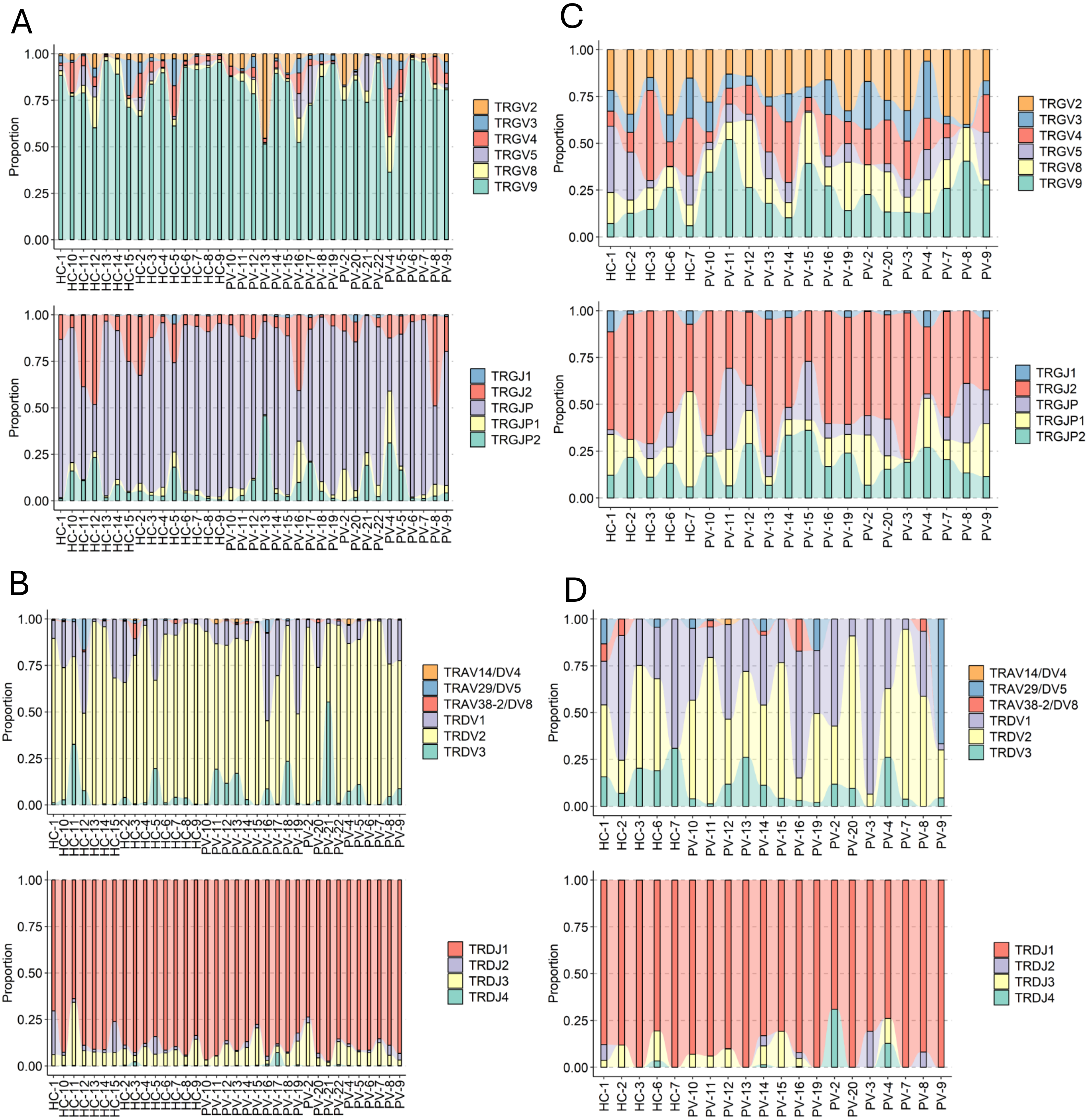

Supplementary Figure 4. Circulating γδ T cell repertoires are dominated by TRGV9/TRGJP and TRDV2/TRDJ1 clonotypes, while cutaneous repertoires exhibit more diverse TRGV/J and TRDV/J gene usage. ***A, C)*** *TRGV and TRGJ usage in peripheral blood (**A**) and skin (**C**).* ***B, D)*** *TRDV and TRDJ usage in peripheral blood (**B**) and skin (**D**).*

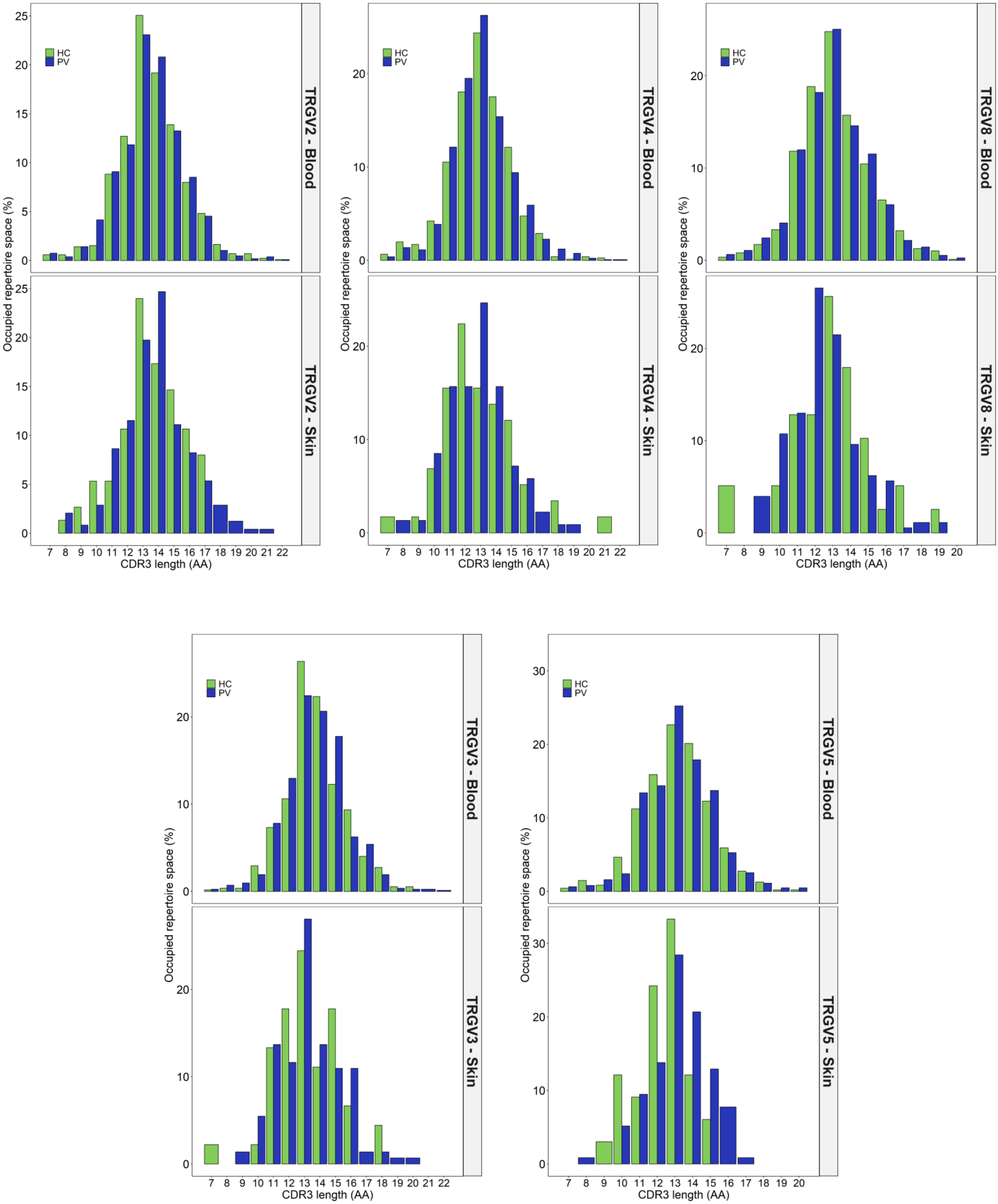

Supplementary Figure 5. Comparable CDR3 length distributions of TRGV2/3/4/5/8 clonotypes in peripheral and cutaneous γδ T cell repertoires. *Data represent the median percentage of repertoire occupancy per CDR3 length for each group: peripheral blood (psoriasis [PV], N = 20; healthy controls [HC], N = 15) and skin (PV, N = 15; HC, N = 5).*

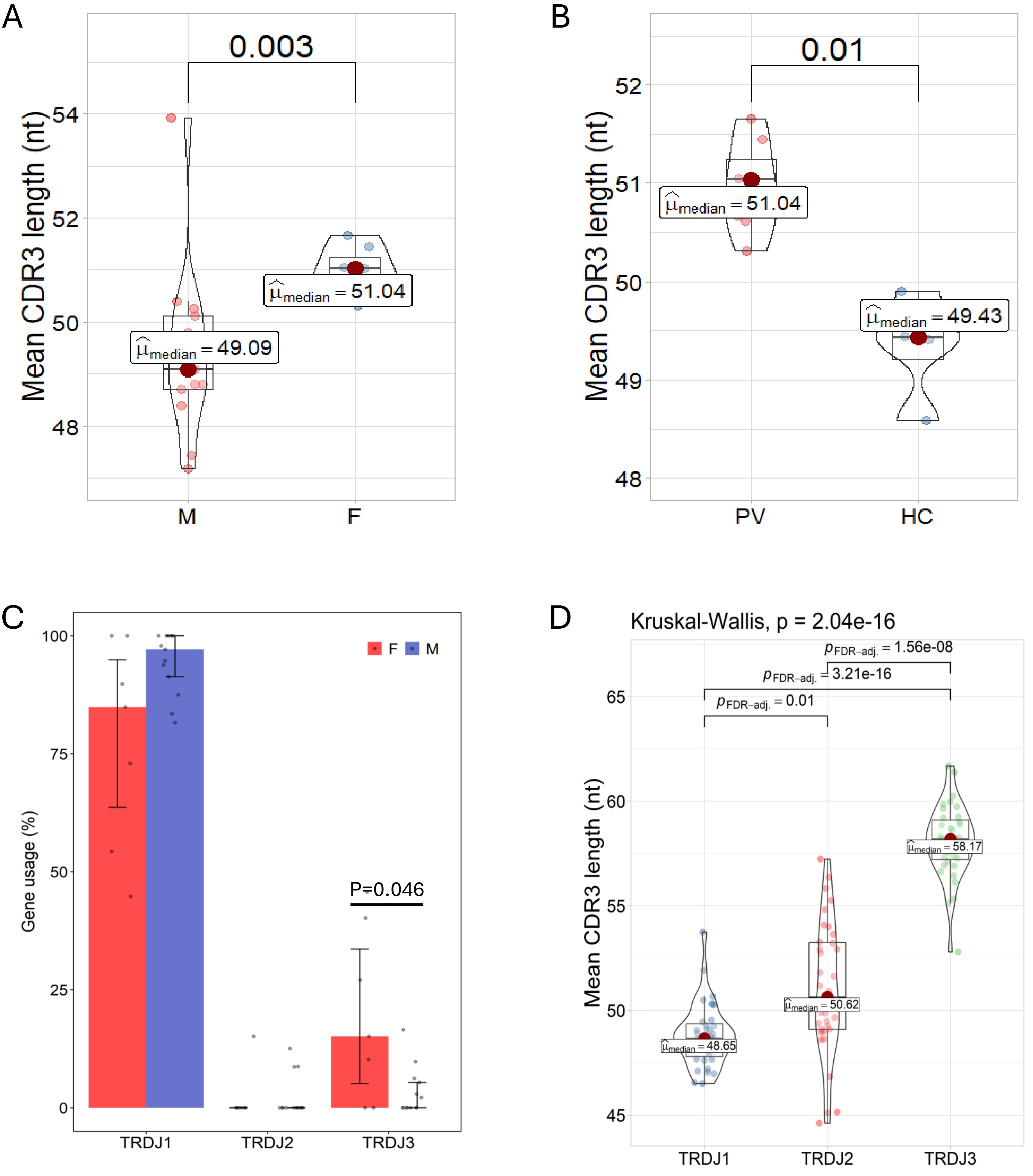

Supplementary Figure 6. Longer Vδ2 CDR3 regions in female psoriasis patients are associated with increased usage of TRDV2-TRDJ3 rearrangements. ***A-B)*** *CDR3δ2 lengths of female psoriasis patients (N = 7) compared to male patients (N = 13) (**A**) and healthy female controls (N = 4) (**B**).* ***C)*** *Proportion of TRDV2-TRDJ3 clonotypes among the top 10 most abundant sequences per repertoire, stratified by sex.* ***D****) Comparative CDR3 lengths of TRDV2 clonotypes using TRDJ1, TRDJ2, or TRDJ3 gene segments. Statistical comparisons in* ***A–C*** *were performed using the Mann–Whitney U test;* ***D*** *was analyzed using the Kruskal–Wallis test with Benjamini–Hochberg correction.*

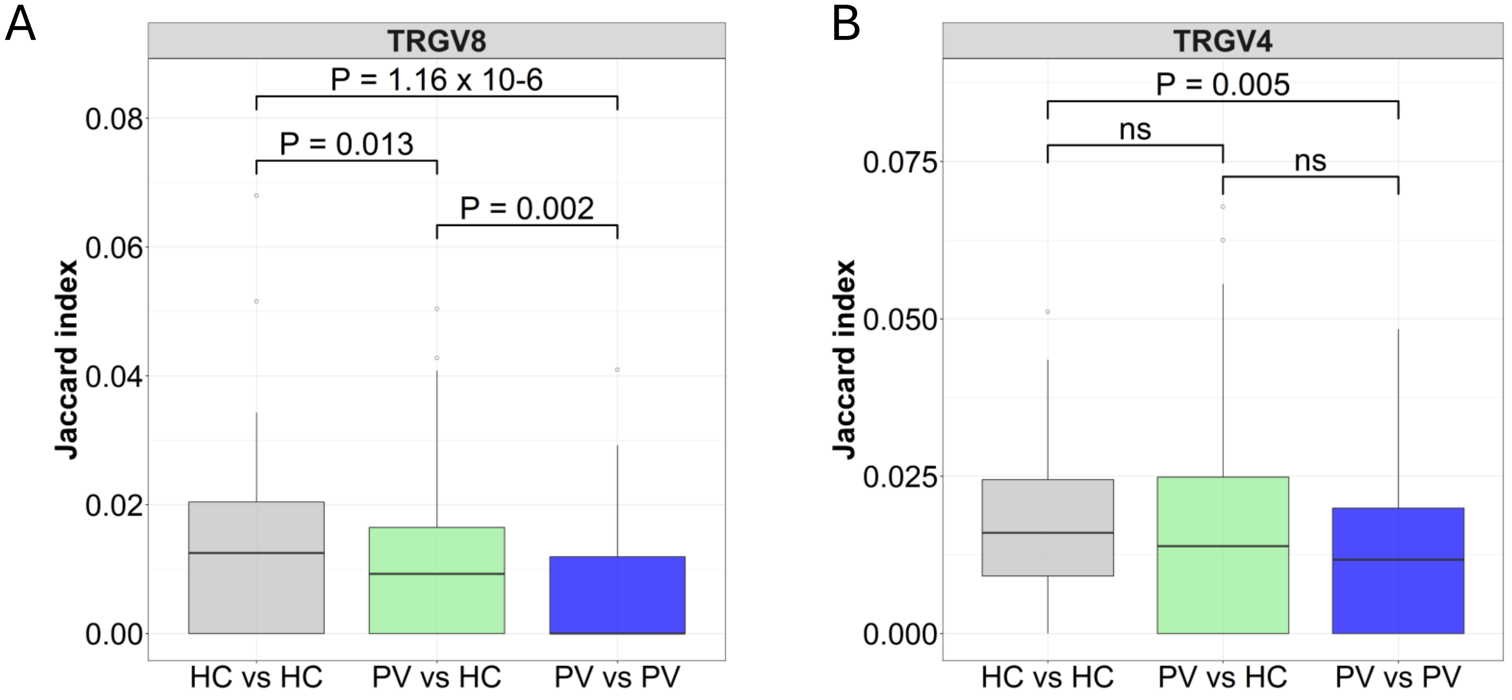

Supplementary Figure 7. Reduced intra-group sharing of TRGV8 and TRGV4 clonotypes in psoriasis patients compared to healthy controls. *For TRGV8 (**A**), overlap is significantly lower within the psoriasis group than within healthy controls, and overlap between PV and HC individuals exceeds that among PV patients themselves, suggesting the persistence of shared "public" clonotypes in health that are diminished or diversified in psoriasis. For TRGV4 (**B**), intra-group overlap is similarly reduced in PV patients, though the difference between patient-control and intra-group comparisons is not significant. Statistical comparisons were performed using the Mann–Whitney U test.*

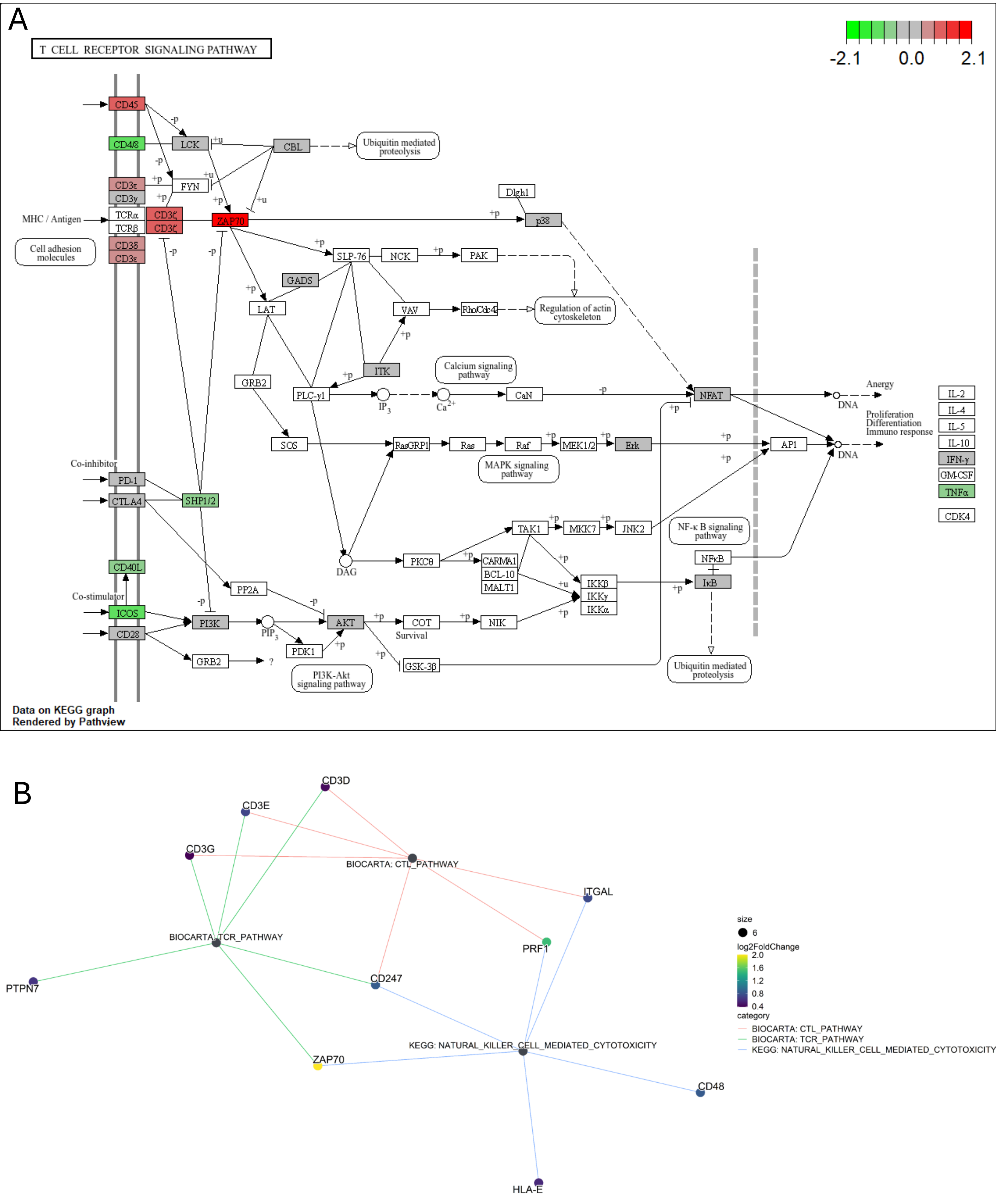

Supplementary Figure 8. Upregulated genes in circulating γδ T cells from psoriasis patients are enriched in T cell receptor signaling pathways. ***A****) KEGG T cell receptor signaling pathway map generated with Pathview, highlighting upregulated (red) and downregulated (green) genes in circulating γδ T cells from psoriasis patients versus healthy controls.* ***B****) cnetplot of GSEA results showing enrichment of upregulated genes in Biocarta TCR signaling, KEGG TCR signaling, and NK cell-mediated cytotoxicity pathways.*

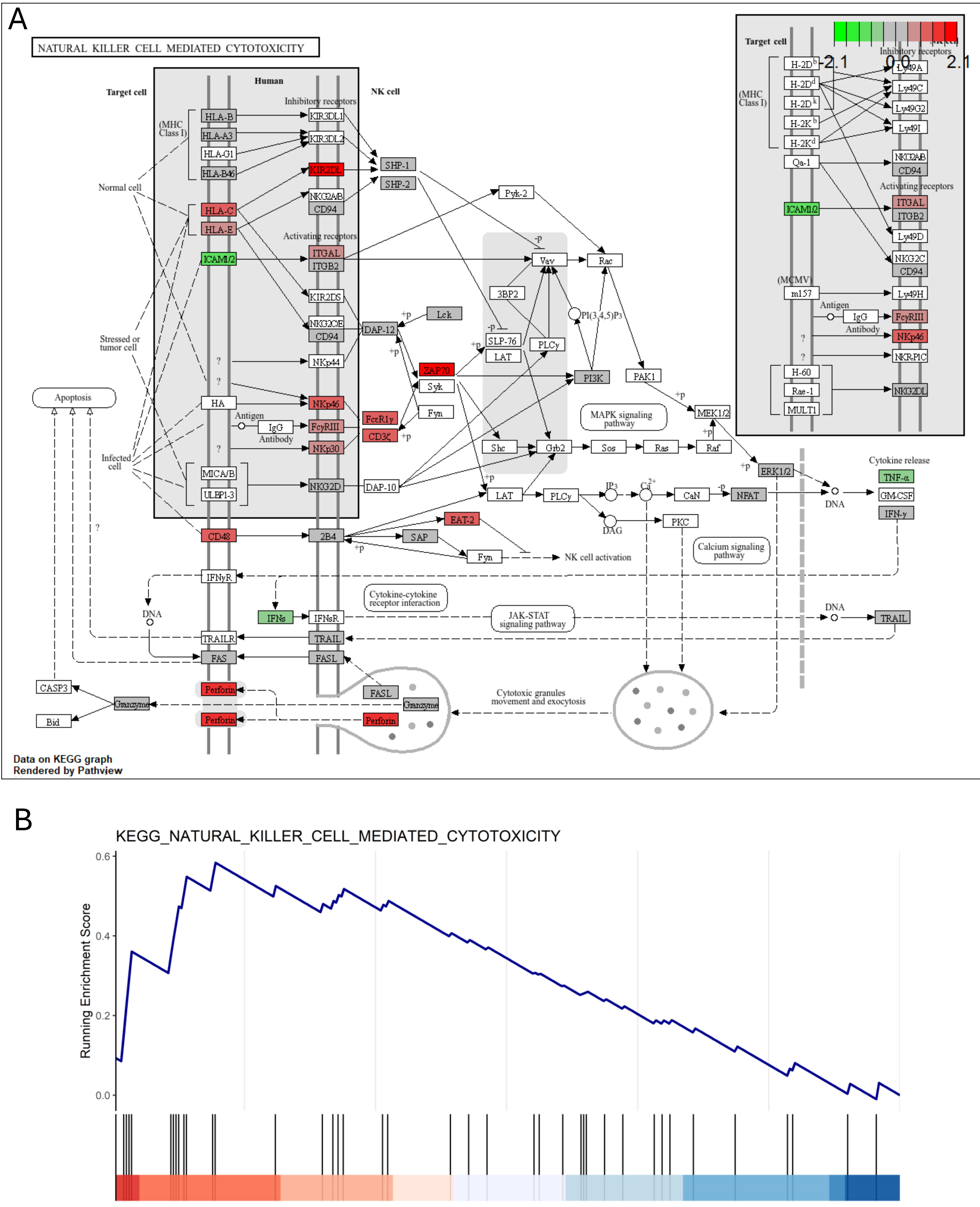

Supplementary Figure 9. Upregulated genes in circulating γδ T cells from psoriasis patients are enriched in cytotoxicity-related pathways. ***A****) KEGG NK cell-mediated cytotoxicity pathway map generated with Pathview, showing upregulated (red) and downregulated (green) genes.* ***B****) GSEA running enrichment score plot demonstrating significant enrichment of genes in the KEGG NK cell-mediated cytotoxicity pathway.*

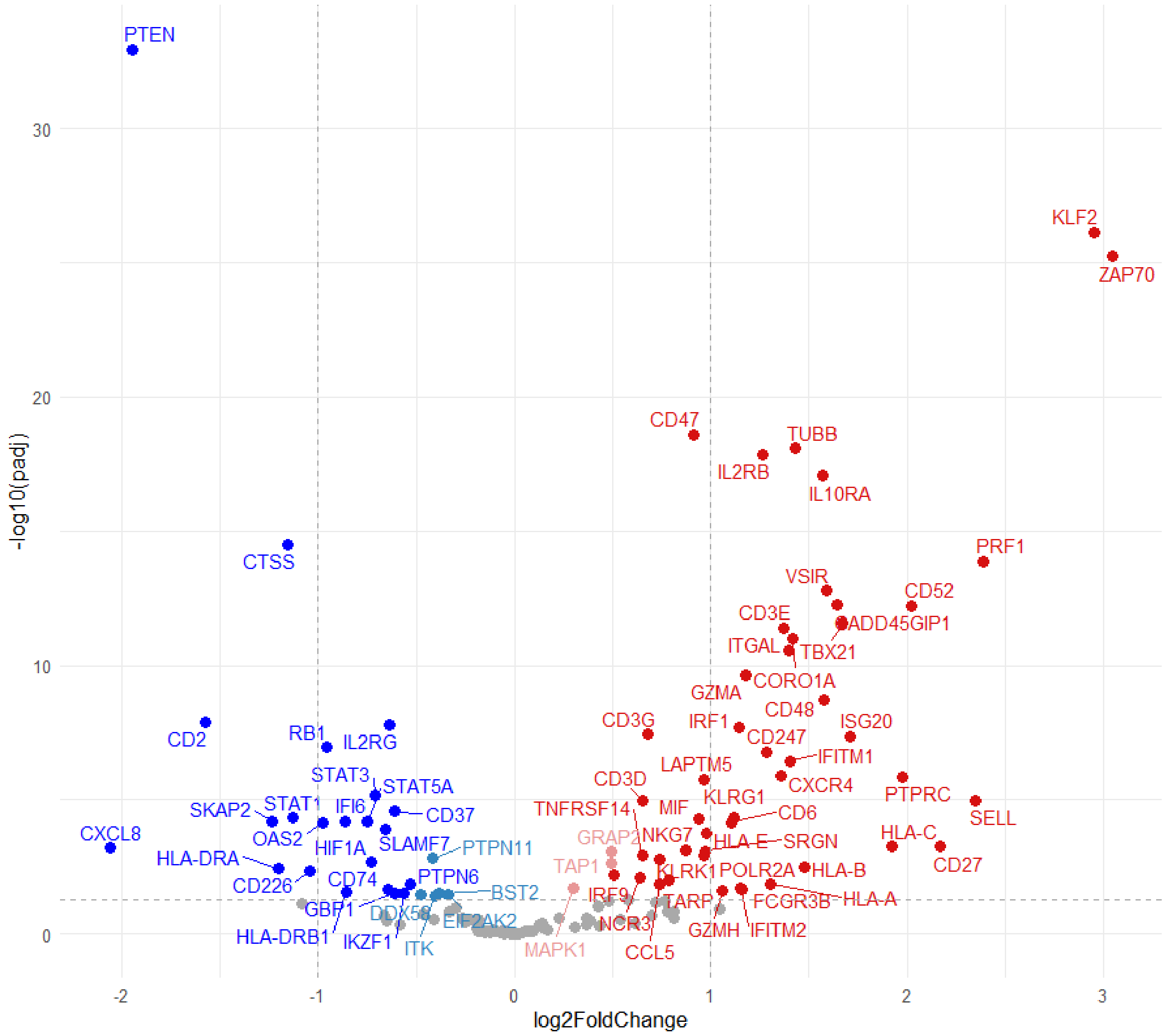

Supplementary Figure 10. Differential gene expression between PV-A subgroup and healthy controls. *Differential expression analysis was performed between PV-A (PV-4, PV-5, PV-6, PV-7, PV-8, and PV-9) and healthy controls (N = 11). A total of 139 significantly modulated genes were identified out of 300 transcripts tested, and transcripts with a baseMean > 500 are shown. Significantly upregulated genes ($log_2FC > 1$, adj. $p < 0.05$) are shown in dark red; moderately upregulated genes ($log_2FC > 0$, adj. $p < 0.05$) in light red. Significantly downregulated genes ($log_2FC < -1$, adj. $p < 0.05$) are in dark blue; moderately downregulated ($log_2FC < 0$, adj. $p < 0.05$) in light blue. Non-significant genes are grey. Dashed lines indicate $log_2FC = \pm 1$ and adj. $p = 0.05$.*
