# Supplementary material for: Psoriasis vulgaris leaves a dynamic imprint on circulating and skin γδ TCR repertoires shaped by disease severity, age, and sex
Source: Front Immunol. 2025 Oct 17;16:1670364. doi: 10.3389/fimmu.2025.1670364 (PMC12576707; doi:10.3389/fimmu.2025.1670364)
Supplement: Supplementary file 2 [file DataSheet2.zip › Supplementary_Tables_1-12_15_16.pdf]

Supplementary Table 1. Antibodies used in γδ T cell immunophenotyping

Antibody	Fluorochrome	Clone	Source / manufacturer	Dilution
CD3	FITC	UCHT1 gamma	Dpt. of Immunology and Biotechnology, University of Pécs, Hungary	1:250
γδTCR	PE-Cy7	B1	BioLegend	1:100
TCR Vδ1	APC	TS8.2	eBiosciences	1:100
TCR Vδ2	PerCP/Cy5.5	B6	BioLegend	1:200

Supplementary Table 2. Comparative metrics of blood γδ TCR sequencing in PV vs HC

	PV (N = 20)	HC (N = 15)	P
TRG reads	631,219 (443,787 – 726,651)	511,311 (346,524 – 571,758)	0.067
TRD reads	248,937 (160,673 – 351,775)	267,126 (169,681 – 294,735)	0.498
TRG/TRD reads (%)	89.2 (84 – 92.7)	81.6 (75.7 – 89.5)	0.116
TRG clonotypes	748 (351 – 1738)	1235 (897 – 1617)	0.170
TRD clonotypes	497 (157 – 1192)	772 (534 – 1224)	0.204

Data are shown as median with interquartile range. Mann-Whitney U test

Supplementary Table 3. Comparative metrics of skin γδ TCR sequencing in PV vs HC

	PV (N = 15)	HC (N = 5)	P
TRG reads	32,388 (21,445 – 41,098)	13,809 (11.665 – 30,678)	0.138
TRD reads	8248 (3430 – 12,041)	5533 (2374 – 5595)	0.337
TRG/TRD reads (%)	1.71 (1.27 – 2.62)	2.49 (1.4 – 4.21)	0.663
TRG clonotypes	101 (77.5 – 115)	91 (72 – 96)	0.222
TRD clonotypes	14 (12.5 – 26)	16 (10 – 17)	0.759

Data are shown as median with interquartile range. Mann-Whitney U test

Supplementary Table 4. Peripheral blood TCRγ and TCRδ sequencing metrics by sample

Sample ID	Status	Sex	Age	PASI	Read count		Unique clonotypes	
					TCRγ	TCR δ	TCRγ	TCR δ
PV-2	PV	F	67	19	795647	119120	118	75
PV-4	PV	F	44	5.8	584535	222040	573	712
PV-5	PV	M	26	7.8	419318	360982	1481	1479
PV-6	PV	M	30	5.1	655658	353850	1870	2147
PV-7	PV	M	22	4	755437	313981	1275	1356
PV-8	PV	M	67	21.6	598269	193791	472	450
PV-9	PV	M	33	24	539348	274515	1184	1130
PV-10	PV	M	68	36.9	583820	376106	194	161
PV-11	PV	M	54	25.2	610873	216030	214	145
PV-12	PV	F	19	22	535212	322799	2095	2168
PV-13	PV	M	44	9	595663	303751	426	474
PV-14	PV	M	22	3.4	666749	346932	2145	2112
PV-15	PV	F	39	20.1	525342	377865	835	917
PV-16	PV	M	34	5.4	226541	153358	1211	854
PV-17	PV	M	37	4.8	374861	164051	500	512
PV-18	PV	F	43	27.9	690506	158570	261	254
PV-19	PV	F	72	7.7	538738	157767	449	423
PV-20	PV	M	36	4	480310	437744	1215	1529
PV-21	PV	F	58	8.1	520844	161227	296	242
PV-22	PV	M	46	12.8	358070	199152	878	961
HC-1	HC	F	37	NA	556551	313410	790	708
HC-2	HC	F	37	NA	428775	281194	845	807
HC-3	HC	F	55	NA	480576	264478	659	528
HC-4	HC	M	33	NA	730404	424444	1466	1755
HC-5	HC	F	34	NA	431006	297172	1005	958
HC-6	HC	M	29	NA	437208	291242	407	433
HC-7	HC	M	29	NA	350440	256246	1217	1429
HC-8	HC	M	37	NA	265632	203702	834	1003
HC-9	HC	M	28	NA	254003	144771	1544	2086
HC-10	HC	M	43	NA	281347	128826	786	761
HC-11	HC	M	33	NA	468200	152641	257	256
HC-12	HC	M	40	NA	269766	94256	605	520
HC-13	HC	M	29	NA	536006	317303	403	436
HC-14	HC	M	34	NA	461820	277604	1518	2072
HC-15	HC	M	23	NA	308416	186134	611	603

Supplementary Table 5. Skin TCRγ and TCRδ sequencing metrics by sample

Sample ID	Status	Sex	Age	PASI	Read count		Unique clonotypes	
					TCRγ	TCR δ	TCRγ	TCR δ
PV-2	PV	M	30	1.8	14413	7357	19	7
PV-3	PV	F	35	16.1	19575	2589	42	5
PV-4	PV	M	57	7.2	5660	1967	32	7
PV-7	PV	M	47	12.5	24190	3343	85	12
PV-8	PV	F	67	19	9515	2773	47	10
PV-9	PV	M	52	31.9	31523	12603	37	15
PV-10	PV	F	38	9.6	24009	7750	88	19
PV-11	PV	M	24	18	17346	10875	68	37
PV-12	PV	M	30	5.1	227670	15716	170	23
PV-13	PV	M	67	21.6	23863	8370	50	11
PV-14	PV	M	33	24	63123	25482	181	55
PV-15	PV	M	66	9	11005	2786	48	9
PV-16	PV	M	38	20.9	25160	29452	112	49
PV-19	PV	F	57	1.2	9557	2581	117	21
PV-20	PV	M	51	2.1	6998	1172	56	8
HC-1	HC	M	34	NA	8633	1903	62	15
HC-2	HC	F	63	NA	5801	3114	34	7
HC-3	HC	F	65	NA	37737	5533	70	17
HC-6	HC	M	55	NA	23272	10729	93	29
HC-7	HC	M	29	NA	12392	1781	45	6

Supplementary Table 6. TRGV / TRGJ segment usage frequencies in circulating repertoires

	PV (N = 20)	HC (N = 15)	P
TRGV9	83.18 (74.19 – 88.22)	88.29 (74.17 – 91.98)	0.559
TRGV4	2.04 (0.49 – 5.38)	3.41 (1.75 – 5.61)	0.317
TRGV2	2.57 (1.14 – 7.03)	2.40 (0.73 – 3.35)	0.359
TRGV8	2.48 (1.24 – 6.08)	2.61 (1.69 – 3.85)	0.726
TRGV3	1.10 (0.35 – 4.11)	0.87 (0.42 – 4.31)	0.751
TRGV5	0.62 (0.19 – 1.66)	0.58 (0.31 – 3.77)	0.726
TRGJP	61.42 (50.66 – 70.74)	68.14 (43.06 – 74.56)	0.803
TRGJ1	16.07 (9.87 – 18.20)	17.63 (15.17 – 24.91)	0.113
TRGJP1	7.88 (4.47 – 15.40)	4.73 (3.28 – 8.46)	0.211
TRGJP2	5.62 (2.36 – 17.85)	6.79 (3.61 – 13.03)	0.494
TRGJ2	0.01 (0 – 0.23)	8.15×10^{-5} (0 – 1.13)	0.692

Data are shown as the median percentage with interquartile range. Mann-Whitney U test

Supplementary Table 7. TRDV / TRDJ segment usage frequencies in circulating repertoires

	PV (N = 20)	HC (N = 15)	P
TRDV2	73.61 (68.97 – 93.36)	87.19 (64.89 – 94.67)	0.881
TRDV1	9.36 (2.43 – 22.38)	8.91 (2.89 – 28.08)	0.751
TRDV3	3.67 (0.55 – 11.16)	1.18 (0.5 – 4.02)	0.516
TRDV5	0.24 (0.13 – 0.46)	0.62 (0.18 – 0.79)	0.167
TRDV8	0.29 (0.04 – 0.61)	0.18 (0.02 – 0.60)	1
TRDV4	0 (0 – 0.06)	0 (0 – 0.02)	0.554
TRDV6	$2x10^{-3}$ (0 – 0.03)	0 (0 – 0.01)	0.628
TRDV7	0 (0 – 0.02)	0 (0 – 0.02)	0.899
TRDJ1	93.40 (90.51 – 94.30)	92.60 (89.47 – 94.26)	0.414
TRDJ3	4.93 (2.75 – 7.32)	4.35 (3.80 – 6.16)	0.907
TRDJ2	1.78 (0.62 – 2)	1.46 (1.36 – 2.62)	0.414
TRDJ4	0.11 (0.07 – 0.20)	0.12 (0.05 – 0.24)	0.665

Data are shown as the median percentage with interquartile range. Mann-Whitney U test

Supplementary Table 8. TRGV / TRGJ segment usage frequencies in cutaneous repertoires

	PV (N = 15)	HC (N = 5)	P	P_{adj}
TRGV9	25.94 (13.76 – 31.18)	12.60 (7.14 – 14.65)	0.034*	0.203
TRGV4	16.66 (7.94 – 21.29)	13.08 (10.53 – 30.92)	0.455*	0.683
TRGV2	23.56 (16.35 – 30.28)	21.71 (15.11 – 34.44)	0.839*	0.839
TRGV8	15.39 (9.69 – 19.66)	11.06 (10.94 – 11.52)	0.109*	0.328
TRGV3	7.54 (5.27 – 15.99)	11.12 (9.74 – 14.35)	0.749*	0.839
TRGV5	9.57 (3.57 – 11.2)	15.53 (4.01 – 25.57)	0.121*	0.589
TRGJP	10.88 (7.07 – 18.81)	2.42 (0 – 7.95)	0.116**	0.554
TRGJ1	0.77 (0.13 – 3.58)	1.78 (0.12 – 7.17)	0.598**	0.663
TRGJP1	10.45 (6.39 – 18.55)	9.96 (9.69 – 21.77)	0.541**	0.663
TRGJP2	19.07 (12.44 – 25.53)	12.19 (11.11 – 18.55)	0.222**	0.554
TRGJ2	55.51 (38.57 – 58.61)	54.32 – 52.32 – 66.92)	0.663**	0.663

*Data are shown as the median percentage with interquartile range. *Student's t-test, ** Mann-Whitney U test. Padj calculated using Benjamini-Hochberg correction*

Supplementary Table 9. TRDV /TRDJ segment usage frequencies in cutaneous repertoires

	PV (N = 15)	HC (N = 5)	P	P_{adj}
TRDV2	45.91 (32-85 – 65.59)	38.41 (17.76 – 49.07)	0.383	0.872
TRDV1	34.66 (19.78)	27.59 (24.79 – 66.51)	0.727	0.872
TRDV3	4.34 (2.53 – 11.50)	19.01 (15.71 – 20.31)	0.029	0.174
TRDV5	0 (0 – 2.93)	0 (0 – 4.33)	1	1
TRDV8	0 (0 – 1.09)	0 (0 – 8.86)	0.484	0.872
TRDV4	0 (0 – 0)	0 (0 – 0)	0.644	0.872
TRDJ1	92.09 (81.96 – 100)	88.12 (87.86 – 100)	1	1
TRDJ3	0 (0 – 8.41)	3.73 (0 – 11.88)	0.641	1
TRDJ2	0 (0 – 1.69)	0 (0 – 0)	0.747	1
TRDJ4	0 (0 – 0.22)	0 (0 – 0)	0.819	1

Data are shown as the median percentage with interquartile range. Mann-Whitney U test. Padj calculated using Benjamini-Hochberg correction

Supplementary Table 10. Comparison of TRG and TRD diversity indices between PV patients and healthy controls

	Index	PV (N = 15)	HC (N = 5)	P
**TRG**	Efron-Thisted	710.5 (394.5 – 1222.5)	784 (610 – 1093)	0.726
	Chao1	704 (392.5 – 1229)	788 (607.5 – 1110.5)	0.726
	D50	0.013 (0.022 – 0.006)	0.015 (0.021 – 0.012)	0.378
	Shannon-Wiener	70.721 (10.030 – 227.070)	81.079 (45.717 – 167.579)	0.494
	Inverse Simpson	19.781 (3.916 – 57.633)	27.168 (14.708 – 47.935)	0.433
**TRD**	Efron-Thisted	796.5 (403 – 1393.75)	780 (515 – 1174.5)	0.764
	Chao1	782.5 (380.75 – 1383.5)	761 (523.5 – 1214)	0.653
	D50	0.026 (0.047 – 0.011)	0.021 (0.029 – 0.011)	0.987
	Shannon-Wiener	131.487 (20.36 – 276.076)	94.991 (57.719 – 221.753)	0.677
	Inverse Simpson	25.669 (8.541 – 57.126)	22.999 (15.638 – 50.238)	0.629

Data are shown as the median percentage with interquartile range. Mann-Whitney U test.

Supplementary Table 11. Significant correlations of PASI scores with diversity indices and clonotype frequency groups in circulating TRG and TRD repertoires

	Index or frequency group	ρ	P
**TRG**	Efron-Thisted	-0.533	0.015
	Chao1	-0.566	0.009
	Shannon-Wiener	-0.493	0.027
	Inverse Simpson	-0.457	0.04
	Small	-0.545	0.013
	Hyperexpanded	0.549	0.012
**TRGV9**	Efron-Thisted	-0.524	0.018
	Chao1	-0.517	0.019
	Shannon-Wiener	-0.475	0.034
	Inverse Simpson	-0.519	0.019
	Small	-0.516	0.020
	Hyperexpanded	0.587	0.006
**TRGV2**	Efron-Thisted	-0.466	0.038
**TRGV8**	Shannon-Wiener	-0.478	0.033
**TRGV4**	Shannon-Wiener	-0.513	0.025
	Inverse Simpson	-0.649	0.003
**TRD**	Efron-Thisted	-0.519	0.019
	Chao1	-0.495	0.027
	Shannon-Wiener	-0.529	0.017
	Small	-0.530	0.016
**TRDV2**	Chao1	-0.529	0.017
	Efron-Thisted	-0.536	0.015
	Shannon-Wiener	-0.490	0.028
	Small	-0.513	0.021
**TRDV3**	Inverse Simpson	-0.603	0.013
	Shannon-Wiener	-0.597	0.015

Data are presented as Spearman correlation coefficients (ρ) with associated p-values. $p < 0.05$ was considered statistically significant.

Supplementary Table 12. Significant correlations of age with diversity indices and clonotype frequency groups in circulating TRG and TRD repertoires of psoriasis patients

	Index	ρ	P
**TRG**	Efron-Thisted	-0.887	4.77×10^{-6}
	Chao1	-0.884	5.66×10^{-6}
	Shannon-Wiener	-0.828	7.57×10^{-5}
	Inverse Simpson	-0.711	0.002
	Small	-0.829	7.16×10^{-5}
	Medium	-0.652	0.006
	Hyperexpanded	0.644	0.007
**TRGV9**	Efron-Thisted	-0.805	1.66×10^{-4}
	Chao1	-0.853	2.71×10^{-5}
	Shannon-Wiener	-0.789	2.77×10^{-4}
	Inverse Simpson	-0.676	0.004
	Small	-0.816	1.16×10^{-4}
	Medium	-0.733	0.001
	Hyperexpanded	0.602	0.014
**TRGV2**	Efron-Thisted	-0.857	2.21×10^{-5}
	Chao1	-0.881	6.45×10^{-6}
	Small	-0.720	0.002
**TRGV8**	Efron-Thisted	-0.832	6.39×10^{-5}
	Chao1	-0.832	6.39×10^{-5}
	Shannon-Wiener	-0.541	0.031
	Inverse Simpson	-0.501	0.048
	Small	-0.797	2.2×10^{-4}
**TRGV3**	Efron-Thisted	-0.635	0.008
	Chao1	-0.689	0.003
	Small	-0.564	0.023
**TRGV4**	Efron-Thisted	-0.652	0.006
	Chao1	-0.668	0.005
	Shannon-Wiener	-0.509	0.044
	Small	-0.626	0.009
**TRGV5**	Efron-Thisted	-0.566	0.022
	Chao1	-0.566	0.022
	D50	0.518	0.039
**TRD**	Efron-Thisted	-0.856	2.37×10^{-5}
	Chao1	-0.841	4.49×10^{-5}
	Shannon-Wiener	-0.800	2.01×10^{-4}
	Inverse Simpson	-0.624	9.72×10^{-3}
	Small	-0.834	6.03×10^{-5}
	Medium	-0.566	0.022
	Hyperexpanded	0.638	0.008

**TRDV2**	Efron-Thisted	-0.822	9.21×10^{-5}
	Chao1	-0.822	7.16×10^{-5}
	Shannon-Wiener	-0.801	1.91×10^{-4}
	Inverse Simpson	-0.669	0.004
	D50	-0.573	0.020
	Small	-0.813	1.29×10^{-4}
	Medium	-0.542	0.03
	Hyperexpanded	0.583	0.018
**TRDV3**	Chao1	-0.619	0.018
	Efron-Thisted	-0.602	0.023
	Small	-0.756	4.58×10^{-4}

Data are presented as Spearman correlation coefficients (ρ) with associated p-values. $p < 0.05$ was considered statistically significant.

Supplementary Tables 13 and 14 are presented in an excel spreadsheet

Supplementary Table 15. Differentially expressed genes in circulating γδ T cells from PV patients

Gene	baseMean	log2FoldChange	pvalue	padj
**ZAP70**	869.6	2.003	6.34E-06	0.001292
**KLF2**	2466.4	1.904	1.84E-05	0.001306
**SELL**	795.6	1.672	0.000445	0.007893
**PRF1**	5356.0	1.490	8.94E-05	0.002851
**CD52**	13914.7	1.145	0.001044	0.014056
**ISG20**	797.0	1.030	0.000367	0.007254
**TBX21**	3084.0	1.005	9.37E-05	0.002851
**GADD45GIP1**	723.8	0.983	0.000152	0.00374
**CD247**	8774.9	0.854	4.42E-05	0.002352
**CD48**	2869.9	0.840	0.00382	0.028057
**CXCR4**	40875.6	0.824	0.001101	0.014056
**POLR2A**	1612.6	0.792	0.000158	0.00374
**TUBB**	740.6	0.763	0.002703	0.024901
**ITGAL**	5055.0	0.747	0.002693	0.024901
**IFITM1**	7952.0	0.727	0.006335	0.037484
**CORO1A**	9285.3	0.719	0.006303	0.037484
**CD3E**	17735.2	0.719	0.002814	0.024901
**IL2RB**	4030.3	0.702	0.000642	0.009769
**IRF1**	2049.7	0.651	0.001774	0.018889
**GZMA**	10056.9	0.649	0.002962	0.024901

**SRGN**	17975.8	0.638	0.001493	0.016736
**PTPN7**	501.8	0.631	0.001188	0.014056
**NKG7**	49905.6	0.572	0.001163	0.014056
**HLA-E**	52623.7	0.564	0.003485	0.026508
**MIF**	2127.3	0.543	0.003368	0.026508
**CD47**	3580.6	0.532	7.4E-05	0.002851
**GPR18**	269.8	0.463	0.004014	0.028496
**CD3D**	10133.9	0.423	0.000485	0.007951
**CD3G**	8183.2	0.382	0.004793	0.031906
**STAT6**	567.3	0.292	0.005842	0.036885
**IL2RG**	10699.2	-0.332	0.005888	0.036885
**MTOR**	338.1	-0.335	0.00304	0.024901
**MX1**	624.5	-0.429	0.002436	0.024707
**OAS3**	256.9	-0.532	0.000375	0.007254
**ICOS**	245.9	-0.876	0.004578	0.031455
**IFI44L**	163.7	-1.622	1.21E-05	0.001292

*Differential gene expression analysis was performed using DESeq2. Only genes meeting the threshold of padj < 0.05 are shown. *padj - Benjamini–Hochberg adjusted p-value.*

Table 16. Frequencies of γδ T cell subsets in PV and healthy control samples included in transcriptomic profiling

	**PV (N = 12)**	**HC (N = 11)**	**P**
Vδ2 of γδ T (%)	74.15 (44.75 – 95.98)	80.6 (68.7 – 88.8)	0.712
Vδ1 of γδ T (%)	9.44 (2.79 – 16.15)	14 (7.58 – 27.15)	0.372
Vδ1$^-$ Vδ2$^-$ of γδ T (%)	6.08 (2.05 – 15.4)	3.98 (2.59 – 5.97)	0.309

Data are shown as median (interquartile range). Mann-Whitney U test
